# A longitudinal epigenome-wide association study of preeclamptic and normotensive pregnancy

**DOI:** 10.1186/s43682-022-00014-w

**Published:** 2023-01-26

**Authors:** Shuwei Liu, Haoyi Fu, Mitali Ray, Lacey W. Heinsberg, Yvette P. Conley, Cindy M. Anderson, Carl A. Hubel, James M. Roberts, Arun Jeyabalan, Daniel E. Weeks, Mandy J. Schmella

**Affiliations:** 1 Department of Human Genetics, School of Public Health, University of Pittsburgh, Pittsburgh, PA, USA.; 2 Department of Biostatistics, School of Public Health, University of Pittsburgh, Pittsburgh, PA, USA.; 3 Department of Health Promotion and Development, School of Nursing, University of Pittsburgh, 440 Victoria Building, 3500 Victoria Street, Pittsburgh, PA 15261, USA.; 4 Martha S. Pitzer Center for Women, Children and Youth, College of Nursing, The Ohio State University, Columbus, OH, USA.; 5 Magee-Womens Research Institute and Foundation, Pittsburgh, PA, USA.; 6 Department of Obstetrics, Gynecology and Reproductive Sciences, School of Medicine, University of Pittsburgh, Pittsburgh, PA, USA.; 7 Department of Epidemiology, School of Public Health, University of Pittsburgh, Pittsburgh, PA, USA.; 8 Clinical and Translational Science Institute, University of Pittsburgh, Pittsburgh, PA, USA.

**Keywords:** Preeclampsia, Pre-eclampsia, DNA methylation, Epigenetics, Hypertensive disorder of pregnancy, Surrogate variable analysis

## Abstract

**Background:**

While preeclampsia (PE) is a leading cause of pregnancy-related morbidity/mortality, its underlying mechanisms are not fully understood. DNA methylation (DNAm) is a dynamic regulator of gene expression that may offer insight into PE pathophysiology and/or serve as a biomarker (e.g., risk, subtype, a therapeutic response). This study’s purpose was to evaluate for differences in blood-based DNAm across all trimesters between individuals eventually diagnosed with PE (cases) and individuals who remained normotensive throughout pregnancy, did not develop proteinuria, and birthed a normally grown infant (controls).

**Results:**

In the discovery phase, longitudinal, genome-wide DNAm data were generated across three trimesters of pregnancy in 56 participants (*n*=28 cases, *n*=28 controls) individually matched on self-identified race, pre-pregnancy body mass index, smoking, and gestational age at sample collection. An epigenome-wide association study (EWAS) was conducted, using surrogate variable analysis to account for unwanted sources of variation. No CpGs met the genome-wide significance *p* value threshold of 9×10^−8^, but 16 CpGs (trimester 1: 5; trimester 2: 1; trimester 3: 10) met the suggestive significance threshold of 1×10^−5^. DNAm data were also evaluated for differentially methylated regions (DMRs) by PE status. Three DMRs in each trimester were significant after Bonferonni-adjustment. Since only third-trimester samples were available from an independent replication sample (*n*=64 cases, *n*=50 controls), the top suggestive hits from trimester 3 (cg16155413 and cg21882990 associated with *TRAF3IP2-AS1/TRAF3IP2* genes, which also made up the top DMR) were carried forward for replication. During replication, DNAm data were also generated for validation purposes from discovery phase third trimester samples. While significant associations between DNAm and PE status were observed at both sites in the validation sample, no associations between DNAm and PE status were observed in the independent replication sample.

**Conclusions:**

The discovery phase findings for cg16155413/cg21882990 (*TRAF3IP2-AS1/TRAF3IP2*) were validated with a new platform but were not replicated in an independent sample. Given the differences in participant characteristics between the discovery and replication samples, we cannot rule out important signals for these CpGs. Additional research is warranted for cg16155413/cg21882990, as well as top hits in trimesters 1–2 and significant DMRs that were not examined in the replication phase.

## Background

Preeclampsia (PE) is a multi-system, heterogeneous syndrome that affects 5–7% of pregnancies and is a leading cause of pregnancy-related morbidity and mortality in the USA [1, 2]. Beyond acute risk, individuals who survive PE may experience lasting impacts as they are 3–4 times more likely to develop chronic hypertension and have double the risk for myocardial infarction or stroke later in life [3]. Despite the short and long-term morbidity and mortality associated with PE, PE cannot be predicted or prevented, and the only “cure” remains the delivery of the dysfunctional placenta [4]. At present, the current management of PE centers around serial monitoring of maternal and fetal status, and management may include the administration of therapeutic interventions such as antihypertensive medications in the setting of severe hypertension, magnesium sulfate for seizure prophylaxis, and corticosteroids to stimulate fetal lung maturity [4]. Timing of delivery is dependent on gestational age, maternal and fetal status, and presence/absence of severe features [4]. The dearth of early diagnosis and clinical management options for PE is a direct result of the limited understanding of its pathophysiology, including a full appreciation of PE subtypes [5], despite decades of research.

DNA methylation (DNAm) is a dynamic regulator of gene expression that may offer potential insight into the pathophysiology of PE. Moreover, DNAm has the potential to serve as a biomarker of PE, including a biomarker of PE risk, PE subtype, disease progression, and therapeutic response. Although the identification of a clinically useful, one-size-fits-all biomarker may not be feasible given the heterogeneity of PE [5], research that seeks to identify DNAm biomarkers in the context of PE is warranted if we can identify biomarkers that have good predictive value, positively impact maternal/fetal outcomes, and are generalizable to large enough subgroups of individuals who develop PE (e.g., early-onset vs. late-onset or angiogenic vs. non-angiogenic) [6].

Several studies demonstrate that DNAm signatures in the setting of PE differ from uncomplicated pregnancy [7–9]. While the majority of prior studies focused on specimen collection and examination of DNAm *after* the presentation of PE symptoms, this study takes a novel approach by looking at DNAm of peripheral blood *across* pregnancy. Not only does this approach offer unique insight into the mechanisms of PE development, but the focus on data collected prior to the onset of symptoms provides information that is more likely to be clinically actionable to prevent PE or identify its onset earlier in pregnancy. Therefore, the purpose of this study was to provide the first-ever evaluation of differences in blood-based DNAm between preeclamptic and normotensive pregnancy across three trimesters of pregnancy.

## Results

### Discovery phase

#### Sample characteristics

The discovery sample consisted of 56 participants (28 PE cases and 28 normotensive controls) who were originally enrolled in the cohort study entitled “Prenatal Exposures & Preeclampsia Prevention Project (PEPP3): Mechanisms of Preeclampsia and the Impact of Obesity” [P01 HD30367], as described in detail in the “Methods and materials” section and Figure S1. For the discovery EWAS, PE cases were 1:1 matched to normotensive controls on self-identified race, pre-pregnancy body mass index (BMI), smoking status (Y/N: self-report of smoking at least 100 cigarettes in a lifetime), and gestational age at sample collection. Participant characteristics by case-control status are reported in Table 1. The sample was relatively young and primarily made up of participants with overweight/obesity who self-identified their race as Black. As expected, individuals with PE had higher blood pressure prior to birth and also birthed their infants at a significantly earlier gestational age compared to controls.

#### EWAS results

**Fig. 1:**
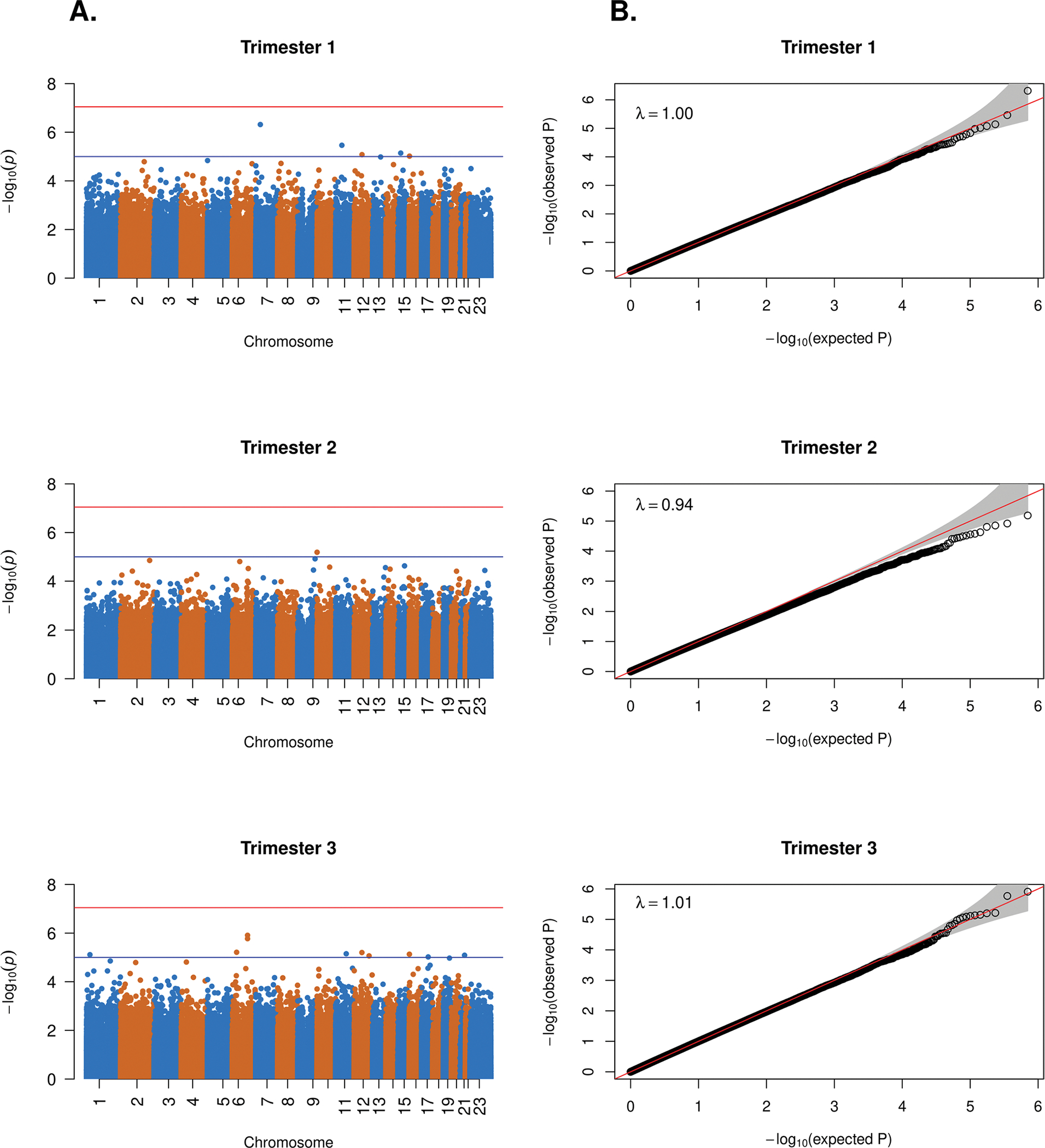
Manhattan and QQ plots for trimesters 1, 2, and 3. **A** Manhattan plots displaying −log_10_(*p* value) on *y*-axis and chromosome number on *x*-axis. The red line represents the threshold for genome-wide significance with a *p* value of 9×10^−8^; the blue line represents the threshold for for suggestive significance with a *p* value of 1×10^−5^. **B** QQ plot with 95% confidence interval displaying observed −log_10_(*p* value) on *y*-axis and expected −log_10_(*p* value) on *x*-axis; the red line represents where the points should fall under the null of no association if the statistical tests are well-calibrated and effects of population substructure and other batch effects are well-controlled for

As detailed below, discovery DNAm data were generated from longitudinally collected peripheral blood samples using the Infinium MethylationEPIC Beadchip. Analyses for the epigenome-wide association study (EWAS) were conducted separately within each trimester. In our analytic model, we regressed DNAm data (*M* values were the outcome variable) on the case-control status and trimester-specific surrogate variables. Surrogate variable analysis (SVA) was applied to account for unwanted sources of variation (i.e., batch effects, cell type heterogeneity) [10]. Final sample sizes after quality control varied across the three trimesters including trimester 1 (50 participants), trimester 2 (53 participants), and trimester 3 (53 participants) as described in the “Methods and materials” section and shown in the Supplementary Materials (Figure S1). In conducting the EWAS, no CpGs reached the genome-wide significant threshold of 9×10^−8^ [11] for trimesters 1, 2, and 3 (Fig. 1A, red line). However, 16 CpGs (5 CpGs in trimester 1; 1 CpG in trimester 2; 10 CpGs in trimester 3) met the genome-wide suggestive significance threshold of 1×10^−5^ (Fig. 1A, blue line).

**Fig. 2:**
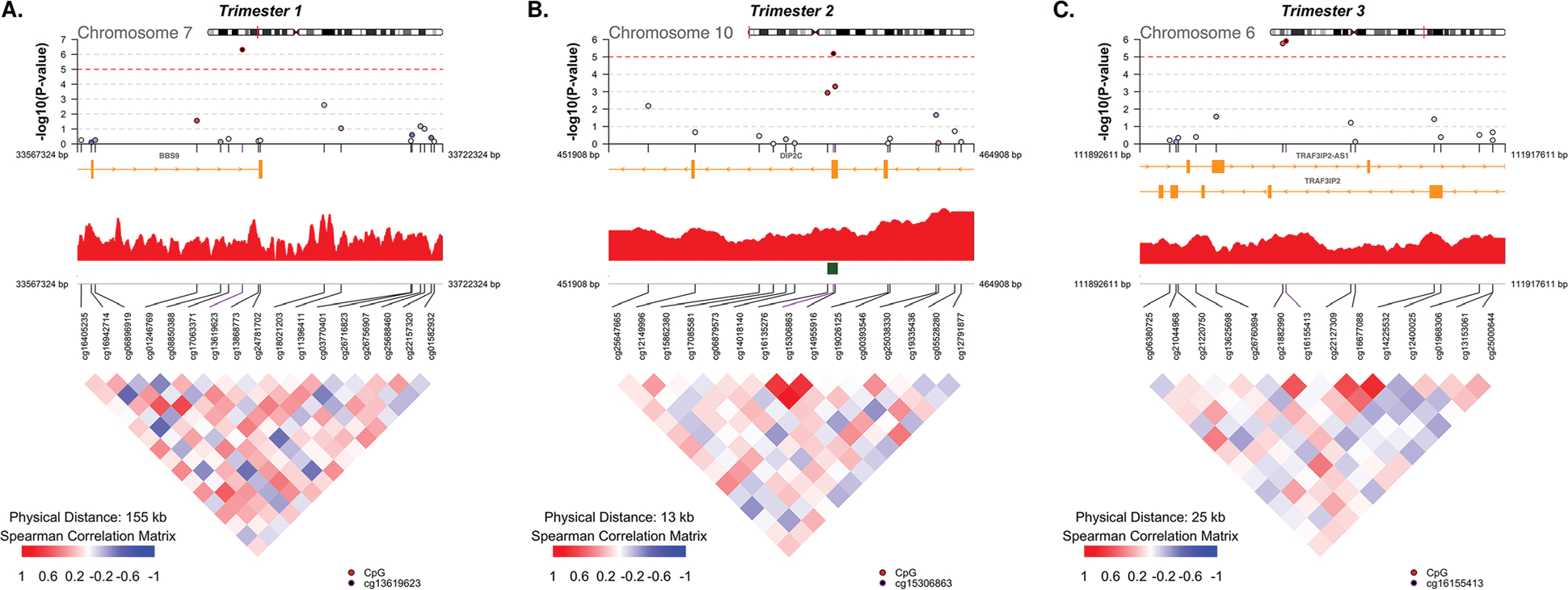
Comet plots of the top CpGs for trimesters 1, 2, and 3. The upper panel of each comet plot shows the EWAS associations with −log_10_(*p* value) on the *y*-axis and CpG position on the *x*-axis; the middle panel depicts annotation tracks, including gene (yellow), GC content (red), and CpG islands (green); the lower panel shows the beta value correlation matrix between the selected CpGs. **A** For trimester 1, a window of length of 15,500 bp on chromosome 7 centered by the top CpG is shown. **B** For trimester 2, there were 15 CpGs within the window of 13,000 bp on chromosome 10 centered by the top CpG. **C** For trimester 3, the window of 25,000 bp on chromosome 6 centered by the top CpG shows 14 CpGs in this region

CpGs with *p* values that met the suggestive significance threshold are detailed in Table 2. QQ plots (which included surrogate variables) for trimesters 1 and 3 revealed that the models accounted for unwanted variation (Fig. 1B, trimesters 1 and 3). However, the QQ plot for trimester 2 indicated some source of unknown/unaccounted variation or simply low power due to the low sample size, as illustrated by the data points that fall below the red diagonal reference line (Fig. 1B, trimester 2); the *p* values for trimester 2 may be a bit conservative (larger than they should be) if the QQ plot pattern is due to unaccounted variation. CoMET plots [12] (plots that display local DNAm correlation patterns and regional EWAS results) centered by the most significant CpG for each trimester are displayed in Fig. 2.

Next, DNAm data were examined for the presence of differentially methylated regions (DMRs) using the dmrff method [13], and results are presented in Table 3. For trimester 1, dmrff identified 3 DMRs on chromosomes 5, 10, and 15. The top region, which maps to *RAB18* on chromosome 10, included 5 CpGs with a Bonferroni-adjusted *p* value of 8.26 ×10^−7^. DMRs on chromosomes 2, 10, and 19 were identified for trimester 2. The most significant hit in trimester 2 contained 8 probes and was located in *NCOA4* on chromosome 10 with a Bonferroni-adjusted *p* value of 9.87 ×10^−4^. For trimester 3, DMRs were identified on chromosomes 6, 14, and 17. The DMR with the lowest Bonferroni-adjusted *p* value of 5.53 ×10^−4^ involved *TRAF3IP2-AS1/TRAF3IP* on chromosome 6; this DMR consisted of the two CpGs which were the top hits for trimester 3 in the discovery EWAS (Table 2, Fig. 2C) and carried forward for replication testing. For each of the 9 DMRs (3 in each trimester) that were significant after the Bonferroni-adjustment, spaghetti plots illustrating DNAm patterns across pregnancy are presented in the Supplemental Materials (Figure S18, S19 and S20).

### Replication phase

#### Sample characteristics

The replication sample was independent of the PEPP3 discovery sample and consisted of 114 participants (64 PE cases, 50 normotensive controls) who were originally enrolled in the first two iterations of the PEPP cohort study (PEPP1 and PEPP2), as described in detail in the “Methods and materials” section and Figure S17. In the replication sample of PEPP1 and PEPP2 participants, only third-trimester DNA samples were available for data collection; we also included, for validation purposes, third-trimester samples from participants included in the discovery EWAS (*n*=53; 25 cases, 28 controls). DNA methylation data were generated (via pyrosequencing as described in detail in the “Methods and materials” section), and replication analyses were performed only for the top suggestive hits from trimester 3 (cg16155413 and cg21882990 located within both *TRAF3IP2* and *TRAF3IP2-AS1*). Data were examined in three stages including (1) validation samples only, (2) independent replication samples only, and (3) combined validation/replication samples. Participant characteristics are described in Table 4.

Participant characteristics in the independent replication sample differed from those of the discovery sample. Specifically, the independent replication sample consisted predominantly of participants who self-identified their race as White (89.1% for PE cases and 90% for normotensive controls), whereas the discovery sample consisted primarily of individuals who self-identified their race as Black (72% for PE cases and 75% for normotensive controls). In addition, participants in the independent replication sample had generally lower mean BMI, earlier gestational age at birth, and greater maternal age compared with individuals in the discovery cohort.

#### Targeted replication results

**Fig. 3:**
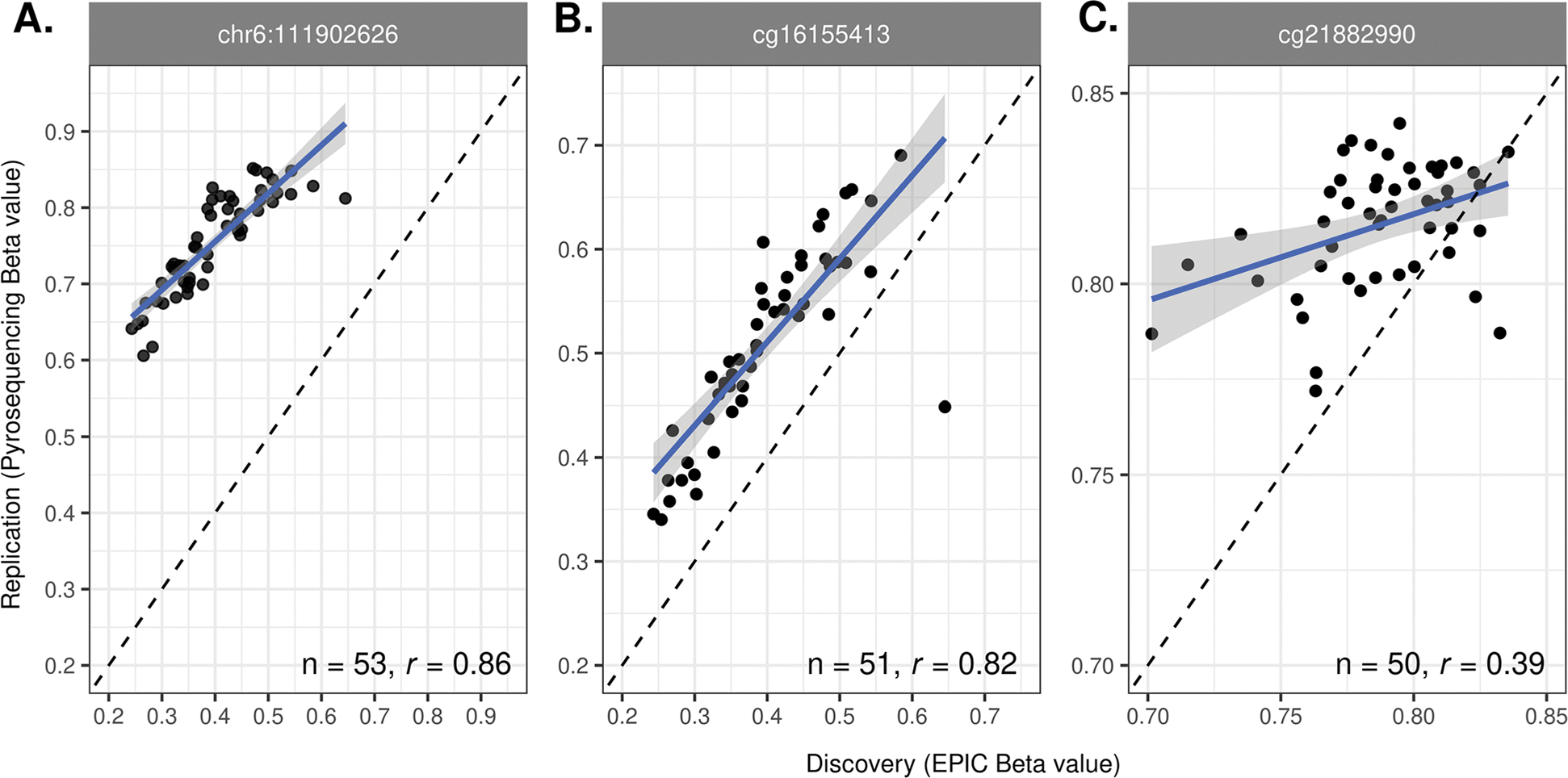
Comparison of DNA methylation beta values for validation samples. *n* is the number of samples and *r* is the Pearson correlation coefficient. The blue solid line is the regression line fitted to the data and the black dashed line is *y*=*x*

In examining the validation samples (i.e., those with DNAm data from both the discovery [Infinium^®^ MethylationEPIC Beadchip] and replication [pyrosequencing] platforms), the DNAm measures derived by these two different technologies were positively correlated as shown in Fig. 3. Both cg16155413 at chr6:111902611 and its adjacent CpG at chr6:111902626 demonstrated stronger correlations (cg16155413: *r*=0.86; chr6:111902626: *r*=0.82) compared to the correlation observed for cg21882990 (*r*=0.39).

**Fig. 4:**
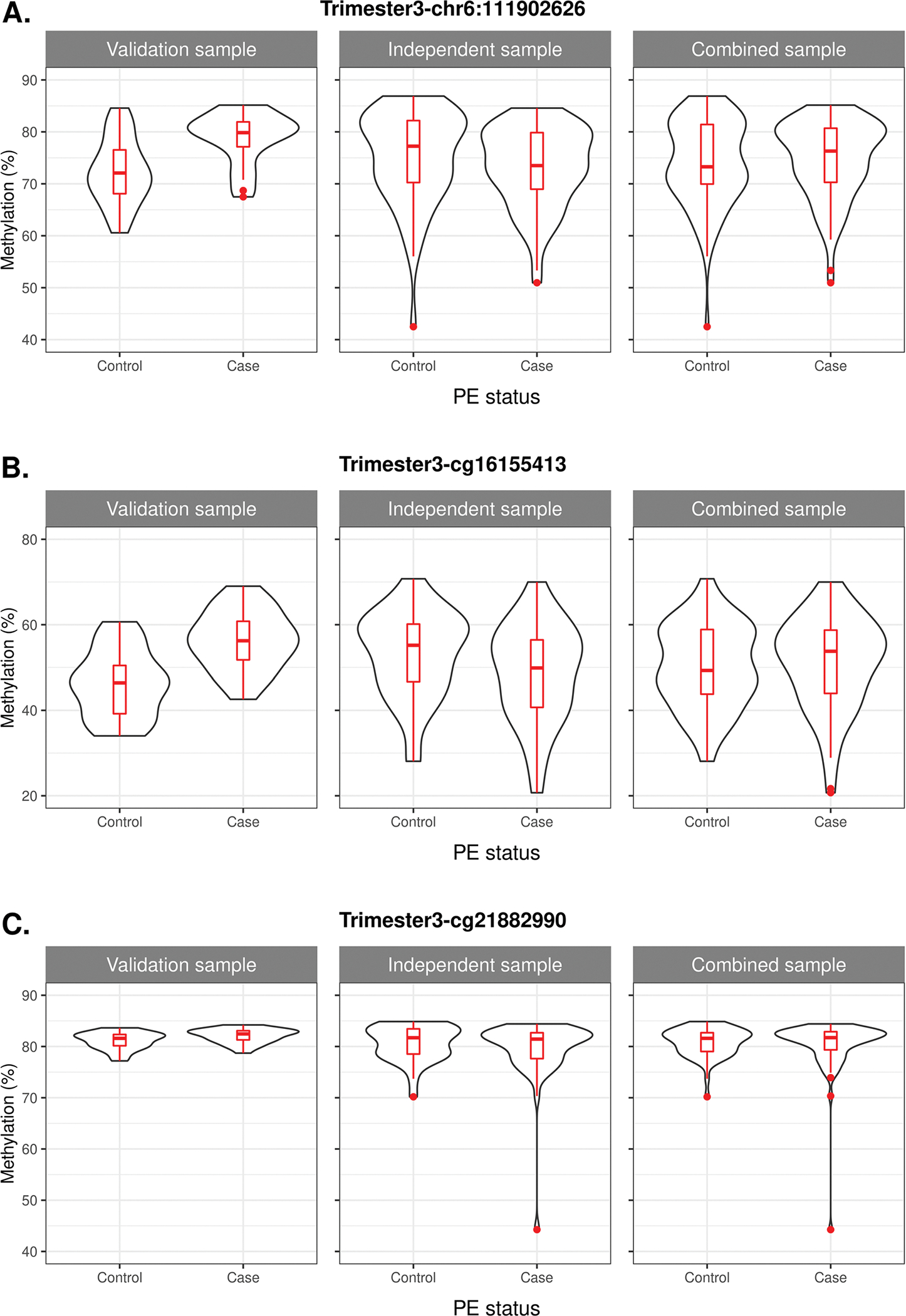
Trimester 3 replication phase: Distribution of DNA methylation levels (as beta values). Validation sample, those with DNAm data from both the discovery (Infinium^®^ MethylationEPIC Beadchip) and replication (pyrosequencing platforms); independent sample, an independent sample of participants completely separate from the discovery phase; combined sample, a combination of validation and independent samples

Replication analyses were performed within three unique groups including (1) validation samples only, (2) independent samples only, and (3) a combined sample of both validation and independent samples. For CpG sites chr6:111902626 and cg16155413, mean methylation *M* values were significantly higher in PE cases at both sites for the validation sample with *p* values of 9.53×10^−5^ and 1.05×10^−4^, respectively (Table 5, Fig. 4, Figure S22). In contrast, in the independent replication sample alone, methylation levels were lower in PE cases than in normotensive controls at both sites, although these differences were not significant (Table 5, Fig. 4, Figure S22). The direction of effect for chr6:111902626 in the combined sample was consistent with the result from the discovery sample (i.e., higher in cases), although the *p* value was not significant and the difference in mean DNAm between PE cases and normotensive controls was small (Table 5, Fig. 4, Figure S22); a similar pattern was observed at cg16155413 in the combined sample. For cg21882990, methylation levels were slightly higher in PE cases for validation samples (consistent with the discovery phase) while it was lower in PE cases for both the replication sample (PEPP1 and PEPP2 participants) and combined sample (Table 5, Fig. 4, Figure S22). Results were similar when we ran linear regression adjusting for race, pre-pregnancy BMI, and maternal age at birth (Table S4).

Finally, as detailed in the “Materials and methods” section, we explored the impact of including additional samples that passed the laboratory quality control but were of uncertain quality (summarized in Table S3, additional samples denoted as “Check” samples) in our analyses. As shown in Table S5, the results were similar with/without the inclusion of these additional samples.

## Discussion

Individuals who develop PE during pregnancy have been shown to have different DNAm levels in the peripheral blood than individuals who remained normotensive [7–9]. The purpose of this study was to address a critical limitation of prior studies by using a longitudinal approach to characterize DNAm profiles in peripheral blood *across pregnancy* in individuals who did and did not go on to develop PE. While the discovery EWAS identified several suggestive hits, the associations did not remain significant after adjusting for multiple testing. Further, top hits selected for follow-up (trimester 3 cg16155413 and cg21882990 near *TRAF3IP2-AS1/TRAF3IP2*) did not replicate in an independent test sample. Importantly, however, even after strict Bonferroni adjustment, the present study identified nine significant DMRs of the genome that should be examined in the context of PE in future studies. These findings are discussed in further detail below.

The discovery EWAS identified statistically suggestive associations between DNAm at CpG sites in 15 genes and pregnancy outcome (Table 2). Of these 15 genes, six (*TRAF3IP2* [14], *NOTCH4* [15], *COQ10A* [16], *AQP11* [17, 18], *SEPN1* [19], and *TFF3* [20]) have previously been linked to biological processes of pregnancy. Furthermore, four of these six genes (*TRAF3IP2* [14], *NOTCH4* [15], *COQ10A* [16], and *SEPN1* [19]) have been implicated in PE. Most notably, umbilical cord DNAm of *TRAF3IP2*, which was also identified as a DMR in maternal blood in the present study, has been associated with early-onset PE [14].

While no significant associations were identified between DNAm and pregnancy outcome after adjustment for multiple testing, the evaluation of DMRs yielded interesting results. The DMR approach allows for CpGs to be grouped together by proximity, rather than testing each CpG independently, making it more powerful [13, 21, 22]. The evaluation of DMRs during trimester 3 identified two CpGs related to *TRAF3IP2/TRAF3IP2-AS1* as differentially methylated (adjusted *p* value of 5.53 ×10^−4^; Table 3). This was the most significant DMR for trimester 3 and included the two CpGs in the *TRAF3IP2/TRAF3IP2-AS1* region that were identified as the top hits in the initial trimester 3 discovery EWAS. *TRAF3IP2* is known to be involved in the immunoregulatory, interrelated IL-17 and TGFβ signaling pathways [23, 24]. *TRAF3IP2-AS1* encodes an anti-sense long noncoding RNA whose role was unknown until very recently. He et al. identified that *TRAF3IP2-AS1* is a regulator of both IL-17A signaling and TRAF3IP2 expression via the recruitment of SRSF10 [25]. Immunomodulation is a crucial aspect of a healthy pregnancy and maladaptive immunomodulation has been previously implicated as a feature of PE pathophysiology [26–28]. While IL-17 and TGFβ are potent inflammatory mediators that have been linked to PE previously, *TRAF3IP2* links these two pathways, within the context of PE pathophysiology. Consistent with our findings, *TRAF3IP2* has previously been found to be differentially methylated in cord blood, with greater mean methylation in neonates birthed by participants with early onset PE than in neonates birthed by normotensive control participants [14]. It is important to note, however, that the discovery signal observed in *TRAF3IP2*/*TRAF3IP2-AS* did not replicate in the independent replication sample. While failure to replicate this result could certainly mean that this finding is a false positive, as discussed below, there were also considerable differences between the discovery and replication sample cohorts that could have contributed to this null finding (e.g., difference in racial distributions in the discovery and replication sample cohorts, as discussed below in the limitations section). No prior research studies were identified that reported links between the remaining DMRs in trimester 3 or in trimester 2 when we conducted PubMed searches within the context of pregnancy (keywords: gene name AND pregnancy) and PE (keywords: gene name AND preeclampsia). Of the three DMRs identified in trimester 1, *CYP1A1* has previously been linked to pregnancy [29–31]. CYP1A1 protein is involved in the synthesis of cholesterol, steroids, and lipids, as well as in drug metabolism; elevated CYP1A1 protein levels have been previously associated with premature birth, intrauterine growth restriction, and placental abruption, but not PE [29–31].

Our study findings add to the small, but growing, collection of EWAS studies conducted in individuals who developed preeclampsia. In 2013, White et al. reported the first EWAS conducted in a cohort of participants who either developed PE or remained normotensive during pregnancy and who were matched for BMI and maternal age (±5 years) [8]. White et al. (2013) identified 997 differentially methylated CpG sites in the peripheral blood collected within 24 h of birth in an exploratory sample size of *N*=28 (*n*=14 PE, *n*=14 normotensive pregnancy) nulliparous participants [8]. Important differences between White et al. (2013) and the present study include (1) the timing of sample collection, (2) the statistical approach, and (3) racial/ethnic makeup of the sample (White et al. included only participants who identified as being of European descent). Specifically, while all the samples collected as part of the White et al. (2013) study were collected within a 24-h window of birth, not all of our third-trimester samples in our discovery sample were collected around the time of birth (data on gestational age at sample collection and gestational age at birth/delivery are presented Table 1). Likewise, the statistical approach used in the present study discovery phase was more complex/rigorous, controlling for unwanted sources of variation, compared with the two-tailed *t* test approach by White et al. (2013) which did not adjust for potential covariates or cell type heterogeneity. Similar to the present study, however, after adjusting for multiple testing, no significant findings remained in White et al. (2013).

The same research group published a second study using the same data set but applying a candidate gene approach in 2016 [9]. This study evaluated 77 CpG sites from 33 candidate genes and identified six differentially methylated genes (*AGT*, *DDAH1*, *CALCA*, *MTHFR*, *POMC*, *PTGS2)*, four of which were validated in a replication cohort (*AGT*, *DDAH1*, *CALCA*, *POMC)* [9]. Interestingly, none of these genes overlap with our suggestive hits, nor our significant DMRs, discussed above (Tables 2 and 3). The present discovery EWAS also differed from Anderson et al. (2014) who reported 207 differentially methylated CpG sites in the peripheral blood collected during the first trimester in an exploratory sample size of *N*=12 (*n*=6 late-onset [>34 weeks] PE, *n*=6 normotensive pregnancy) nulliparous participants [7]. This pilot study employed a statistical approach similar to White et al. (2013), utilizing a two-tailed *t* test, unadjusted *p* value threshold of 0.05, and did not adjust for potential covariates or cell type heterogeneity. We believe the differences in our findings are likely related to discordant study design including the timing of sample collection and statistical approach.

### Strengths and limitations

There are several strengths to this study, including its longitudinal approach, focus on the peripheral blood, comprehensive phenotyping including 1:1 matching in case/control selection, stringent data QC and analytical procedures that considered cell type heterogeneity and multiple testing, and attempted replication. Despite these strengths, important limitations should be noted. Although the sample size is the largest to date for evaluating blood-based DNAm in PE, important signals in the data could be missed given the stringent approach in correcting for multiple testing. Further, while the discovery sample was primarily comprised of participants who self-identified as Black (which, taken alone, focuses on a historically understudied population, and represents an important strength of the discovery analyses), the replication sample consisted of individuals who primarily self-identified as White. Despite the well-documented differences in DNAm by the self-reported race [32, 33], there were no significant race-specific differences in DNAm of cg16155413 or cg21882990 (data not shown). Moreover, replication analyses could be performed for trimester 3 only. Future work should include the examination of top hits identified in trimesters 1 and 2. Another limitation to note is that the discovery-based EWAS and the targeted replication-based analyses did not utilize analogous analytic approaches with respect to adjustment for cell type heterogeneity. Future collection of genome-wide DNA methylation data in the replication sample (PEPP1 and PEPP2 participants) would allow us to evaluate the relationship between pregnancy outcome and *TRAF3IP2*/*TRAF3IP2-AS* cg16155413 or cg21882990 while accounting for cell type heterogeneity in the replication sample. Finally, it is well recognized that PE is a heterogeneous syndrome, and although individuals who develop PE may exhibit similar manifestations (e.g., hypertension, proteinuria), there are likely numerous PE subtypes that are driven by differing biological mechanisms (e.g., early-onset vs. late-onset) [5]. This complexity adds to the difficulty in identifying one-size-fits-all biomarkers, including biomarkers of disease risk, diagnosis, disease progression, and therapeutic response [5]. Moreover, grouping all individuals who develop PE into one “case” group, as we did in our small study, may impact our ability to fully comprehend subtype pathophysiology, as well as identify biomarkers for different PE subtypes [5]. Ultimately, future research cohorts will need to be large enough to account for PE heterogeneity (e.g., stratification by PE subtype), which will allow us to investigate differing types (e.g., PE risk and disease progression) of shared and subtype-specific biomarkers of PE.

## Conclusion

We did not identify significant differences when comparing DNAm of individual CpG sites in the peripheral blood in individuals who developed preeclampsia to individuals who remained normotensive, across pregnancy. However, even after strict adjustment for multiple testing, we identified three DMRs in trimesters 1, 2, and 3, for a total of 9 DMRs that warrant attention in the future. Of these regions, one mapped to *TRAF3IP2-AS1/TRAF3IP2*, which was also a top suggestive hit in our analysis of individual CpG sites. Of note, our stringent approach was designed to assess for differences that would be meaningful to the pathophysiology of PE. However, future analyses could be conducted with the intention of searching for DNAm patterns which may serve as a biomarker (e.g., not adjusting for cell type heterogeneity) [34, 35].

## Methods and materials

### Participants

#### Discovery sample

Participants included in the discovery EWAS were originally enrolled in the NICHD-funded cohort study entitled “Prenatal Exposures & Preeclampsia Prevention Project (PEPP3): Mechanisms of Preeclampsia and the Impact of Obesity” [P01 HD30367] as detailed elsewhere [32, 36]. In brief, the PEPP3 cohort participants were recruited at the UPMC Magee-Women’s Hospital between 2008 and 2014 to investigate factors that are associated with obesity and PE. Participants were not eligible if they were <14 or >40 years of age and had a multi-fetal pregnancy or history of medical conditions associated with an increased risk of PE (e.g., chronic hypertension, diabetes, chronic renal disease). For the current EWAS study, samples and clinical/demographic data that were collected as part of the longitudinal arm of PEPP3 were used, which included peripheral blood samples from trimesters 1, 2, and 3 of pregnancy [32, 36]. All participants provided written informed consent for their participation in PEPP3 and for the use of their de-identified data and samples in future studies.

As shown in Figure S1, the current EWAS study included 56 PEPP3 participants (28 diagnosed with PE [cases] and 28 who experienced an uncomplicated, normotensive pregnancy outcome [controls]). In designing this EWAS, cases and controls were 1:1 matched based on self-identified race, pre-pregnancy BMI, self-reported smoking status, and gestational age at sample collection (±2 weeks when possible).

#### Replication sample

To support both validation and independent replication of findings, replication phase DNAm data were generated on a new data collection platform for (1) all trimester 3 participants (from the PEPP3 cohort as described above) and (2) an independent sample of participants. Participants in the independent sample were identified from the PEPP1 and PEPP2 cohorts, which were independent of the PEPP3 cohort. PEPP1-2 participants were recruited at the UPMC Magee-Women’s Hospital between 1997 and 2007 either at ≤20 weeks of gestation or at labor/birth due to suspected PE [37]. Because the majority of PEPP1-2 participants who were diagnosed with PE were enrolled in a cross-sectional study arm (recruited in the labor/delivery unit), the replication analyses were restricted to DNA samples collected during the third trimester of pregnancy. Exclusion criteria were the same as described for PEPP3. All participants provided written informed consent for their participation in PEPP1-2 and for the use of their de-identified data and samples in future studies.

As shown in Figure S17, 154 independent PEPP1-2 participants (77 diagnosed with PE [cases] and 77 who experienced an uncomplicated, normotensive pregnancy outcome [controls]) were selected for the replication phase. In designing the replication sample, cases and controls were 1:1 matched based on self-identified race, pre-pregnancy BMI, and gestational age at sample collection (±2 weeks when possible). Unfortunately, 39 samples were subsequently excluded due to a misunderstanding of the gestational age at the sample collection variable and 1 sample was excluded due to misclassified pregnancy outcome. This exclusion interfered with the matched study design for the independent replication phase and resulted in only partial matching (handled statistically as described below). A total of 53 validation samples (PEPP3/trimester 3 discovery sample, 25 cases/25 controls) and 114 independent samples (PEPP1-2, 64 cases/50 controls) were sent for data collection (Figure S17).

### Pregnancy outcome phenotypes

Pregnancy outcome classification was based on a rigorous review of clinical data and adjudication by an expert panel of clinicians and researchers. For both the discovery and replication samples, the PE phenotype was classified as new-onset gestational hypertension and proteinuria in a previously normotensive participant after 20 weeks of gestation. Gestational hypertension was defined as an increase in BP to systolic BP ≥140 mmHg and/or diastolic BP ≥90 mmHg that returned to baseline by 12 weeks postpartum. The average of the last four BPs taken in the labor and delivery suite prior to birth or any therapeutic interventions that would impact blood pressure (e.g., antihypertensive therapy or epidural) was used to determine the presence of gestational hypertension. Proteinuria was defined as either (1) ≥300 mg/24 h, (2) ≥0.3 protein/creatinine ratio, (3) ≥2+ on a random urine dipstick, or (4) ≥1+ on a catheterized urine specimen. Of note, in PEPP1-2 (i.e., replication phase), a diagnosis of PE also required evidence of the following: (1) incremental blood pressure change (increase in systolic BP > 30 mmHg and/or increase in diastolic BP > 15 mmHg based on a comparison of the average of the last four BPs taken in the labor and delivery suite prior to birth or any therapeutic interventions that would impact blood pressure to the average BP prior to 20 weeks of gestation; the number of BPs included in the average blood pressure calculations prior to 20 weeks of gestation was based on the number of prenatal visits prior to 20 weeks gestation that were recorded for each participant) and (2) hyperuricemia (serum uric acid concentration >1 standard deviation from normal for gestational age [38]). The incremental BP changes and hyperuricemia were not required as part of the diagnostic criteria for PE cases in the discovery sample. The control phenotype was classified as a clinically evaluated participant who remained normotensive throughout pregnancy, did not develop proteinuria, and birthed a normally grown infant.

### Biospecimen sampling and DNA extraction methods

Peripheral blood samples were collected via EDTA plasma tubes. DNA was isolated/extracted from white blood cells using protein precipitation methods. DNA samples were stored in 1X TE buffer at ≤−40°C until DNAm data collection.

### Discovery sample DNAm data collection

Fifty-six participants were identified for inclusion in the discovery of EWAS. An overview of the sample selection and elimination process is detailed in the supplement (Figure S1). Genome-wide DNAm data were generated using the Infinium MethylationEPIC Beadchip (Illumina, San Diego, CA, USA) at Johns Hopkins University Genetics Resources Core Facility (*RRID:SCR_018669*). To guard against potential batch effects, an attempt was made to place longitudinal samples from each participant on the same chip and balance the row and column effects as detailed elsewhere [32, 36]. After data processing, DNAm β values and *M* values were calculated as follows:

$$\beta =\frac{\;Meth}{Meth+Unmeth+offset}$$

$$M=\log_{2}\left(\frac{\beta }{1-\beta }\right)=\log_{2}\left(\frac{Meth}{Unmeth+offset\;}\right)$$

where Meth and Unmeth represent methylated and unmethylated signals [39]. The offset term was set to 100 to avoid the problem when the unmethylated value is zero. As M values are more suitable than [0,1]-bounded beta values for statistical analyses, M values were used in all models.

### Discovery sample DNAm data quality control

DNAm QC procedures (described in detail in the supplement) were carried out in R using minfi, lumi, ENmix, and funtooNorm packages [39–44]. Beta values were normalized using funtooNorm functional normalization while using trimester-specific quantile to allow methylation patterns to differ across trimesters. QC procedures included the removal of probes with a detection *P* value >0.01, probes with known SNPs, cross-reactive probes, and Y chromosome probes (since all participants were female) across all participant samples, leaving 703,200 CpG sites for analysis (Table S1). The final sample consisted of 156 unique samples equating to 50 participants in trimester 1, 53 participants in trimester 2, and 53 participants in trimester 3 (Figure S1). Of those 156 samples, 45 participants had measures at all three trimesters, 10 participants had measures at two trimesters, and 1 participant had measures at only a single trimester (Figure S2).

### Replication sample DNAm data collection

For the replication sample, DNAm data were generated via pyrosequencing for top hits in trimester 3 (cg16155413 and cg21882990). Pyrosequencing data were collected at Johns Hopkins University Genetics Resources Core Facility (*RRID:SCR_018669*) using Pyro-Mark CpG assays designed using the automated CpG assay design tool located on the Qiagen^®^ website (Gene-globe catalog #s: cg16155413: PM00681443 [amplicon length: 188 base pairs]; cg21882990: PM00681415 [amplicon length: 143 base pairs]). Prior to pyrosequencing, bisulfite conversion of DNA samples (500 ng converted/sampled) was completed using the Qiagen^®^ EpiTect Bisulfite kit (catalog numbers 59104 and 59110). Pyrosequencing was carried out using the Qiagen^®^ Pyromark Q48 instrument, using standard protocols. This approach not only captures target CpG sites, but also neighboring CpGs. Data collection procedures returned data for two sites at/near cg16155413 and one site at cg21882990 (Table S2). Pyrosequencing data were manually reviewed for quality by laboratory staff using Qiagen Q48 software version 2.4.2 and assigned a “Pass,” “Check,” or “Fail” designation (Table S3, Figure S17). Samples that were given the “Fail” designation were discarded from analyses, and “Check” samples (Table S3) were explored in sensitivity analyses as detailed below (Table S5).

### Statistical analyses

#### Discovery phase epigenome‑wide association study (EWAS)

Within each trimester, association analyses were performed by regressing DNAm data (as M values) on the case-control status and trimester-specific surrogate variables using an empirical Bayes approach, which computes the *p* values using moderated t-statistics [38, 45]. As described above, SVA adjusts for unwanted variation due to cell type heterogeneity, batch effects, and other possible sources [10, 46, 47]. To interpret results, regional plots around the top CpG for each trimester were drawn using the coMET R package [12].

Next, DMRs were identified using the dmrff R package, which combines EWAS summary statistics from nearby CpG sites [13]. DMRs were characterized by DNAm that was consistently associated with PE status across several CpGs in a local region. Parameters used in the DMR analysis included (1) the distance between two successive sites could not exceed 500 bp, (2) the genomic regions must have had EWAS nominal *p* values of less than 0.05, and (3) the EWAS effect estimates of individual CpG sites in each 500 bp window of the DMR were required to have the same sign [13, 48]. *P* values for each DMR were then Bonferroni-adjusted to correct for multiple testing where the number of tests is equal to the total number of EWAS tests and sub-region tests [13].

#### Replication analysis

Replication analyses were performed independently within three groups including (1) validation samples (i.e., those with DNAm data available from both the discovery and replication platforms) only, (2) independent samples only (i.e., those from a completely new set of participants independent of the discovery phase), and (3) a combined sample of both validation and independent replication samples. These analyses were performed using the partially overlapping samples *t* test [49] as implemented in the Partiallyoverlapping R package [50] to assess the difference in *M* values by case-control status. The partially overlapping samples *t* test is a generalized form of the traditional two-sample *t* test with the added benefit of reducing bias in samples containing both matched/unmatched participants [49, 51]. Only samples with pyrosequencing data that passed laboratory QC checks (i.e., a “Pass” laboratory designation) were used in the main analysis, and samples with uncertain quality that did not fail the laboratory QC checks (i.e., a “Check” laboratory designation, Table S3) were included in a sensitivity analysis. Note that while cell type heterogeneity adjustment during the discovery EWAS was made using the SVA method, this approach could not be carried forward to replication analyses as genome-wide DNAm data were not available.

## Supplementary Material

AdditionalFile1

## Figures and Tables

**Table 1:** Demographic and clinical characteristics of participants in the discovery phase

**Characteristics**	**Preeclampsia cases**	**Normotensive controls**	***p***
**Matched variables**	***n*=28**	***n*=28**	
Self-identified race, *n* (%)			
*Black*	21 (75.0%)	21 (75.0%)	NA*
*White*	7 (25.0%)	7 (25.0%)	
Pre-pregnancy BMI, mean kg/m^2^ (SD)	33.0 (7.5)	33.7 (7.5)	
Lifetime smoking status (no), *n* (%)	18 (64.3%)	19 (67.9%)	
Gestational age at sample collection, mean weeks (SD)			
*Trimester 1*	8.7 (1.7)	8.8 (1.6)	
*Trimester 2*	19.8 (1.7)	19.6 (0.8)	
*Trimester 3*	37.0 (2.5)	37.4 (2.5)	
	**Preeclampsia cases**	**Normotensive controls**	***p*** **
**Unmatched variables**	***n*=28**	***n*=28**	
Parity, *n* (%)			
*Nulliparous (Yes)*	21 (75.0%)	24 (85.7%)	0.38
*(No)*	7 (25.0%)	4 (14.3%)	
Maternal age at birth, mean years (SD)	23.9 (5.0)	23.7 (4.2)	0.83
Gestational age at delivery, mean weeks (SD)	37.4 (2.5)	39.5 (1.3)	3.36 ×10^−4^
Blood pressure < 20 weeks, mean mmHg (SD)			
*Systolic*	112.8 (7.3)	111.5 (8.9)	0.63
*Diastolic*	70.1 (6.0)	66.8 (6.1)	0.08
Blood pressure prior to delivery, mean mmHg (SD)			
*Systolic*	148.3 (6.9)	124.5 (6.1)	2.67 ×10^−14^
*Diastolic*	90.5 (8.1)	71.1 (6.2)	2.21 ×10^−11^

*SD* Standard deviation

* Variables matched based on self-identified race, pre-pregnancy BMI, self-reported smoking status, and gestational age at sample collection (± 2 weeks when possible) so *p* values are not reported

** *p* values from paired *t* test comparing groups for continuous variables and exact McNemar’s test for dichotomous variable

**Table 2:** CpGs suggestively associated with preeclampsia status with p values less than 1×10^−5^ from the discovery EWAS

Tri.	Chr.	Position*	Gene name	CpG	Mean DNA methylation as beta-values (%)		Mean DNA methylation as *M* values		Direction (cases vs. controls)	Raw value**	FDR Q value
					Controls	Cases	Controls	Cases			

1	7	33637324	*BBS9*	cg13619623	62.02	65.68	0.71	0.94	+	4.82×10^−7^	0.34
	11	45628269	*N/A*	cg27232360	15.76	17.15	–2.43	–2.28	+	3.43×10^−6^	0.99
	12	57388441	*GPR182*	cg02305251	74.39	71.34	1.55	1.32	–	8.34×10^−6^	0.99
	15	41785772	*ITPKA*	cg25609143	4.23	3.94	–4.52	–4.62	–	7.20×10^−6^	0.99
	16	3151202	*N/A*	cg08717632	75.82	78.43	1.65	1.87	+	9.63×10^−6^	0.99
2	10	459908	*DIP2C*	cg15306863	63.82	70.83	0.84	1.29	+	6.49×10^−6^	1.00
3	1	26127185	*SEPN1*	cg21187265	12.58	10.95	–2.81	–3.03	–	7.73×10^−6^	0.67
	6	111902385	*TRAF3IP2-AS1; TRAF3IP2*	cg21882990^^^	77.24	80.11	1.77	2.02	+	1.70×10^−6^	0.60
	6	111902611	*TRAF3IP2-AS1; TRAF3IP2*	cg16155413^^^	35.16	44.07	–0.90	–0.35	+	1.23×10^−6^	0.60
	6	32163533	*GPSM3; NOTCH4*	cg13721764	3.64	2.83	–4.82	–5.11	–	6.14×10^−6^	0.67
	11	77300361	*AQP11*	cg16768953	5.16	4.55	–4.21	–4.41	–	7.09×10^−6^	0.67
	12	56660492	*COQ10A*	cg00025436	5.00	3.94	–4.35	–4.62	–	6.30×10^−6^	0.67
	12	108992148	*TMEM119*	cg02768162	47.25	51.09	–0.16	0.06	+	8.65×10^−6^	0.67
	16	2298791	*ECI1*	cg19713585	48.56	44.84	–0.08	–0.32	–	7.40×10^−6^	0.67
	17	49198586	*SPAG9*	cg23966705	3.57	3.69	–4.83	–4.72	+	9.56×10^−6^	0.67
	21	43735722	*TFF3*	cg05671561	74.03	76.29	1.51	1.69	+	8.16×10^−6^	0.67

Table ordered by trimester, chromosome, and position; FDR *Q* value indicates the expected proportion of false positives among all significant probes

*Chr.* chromosome, *Tri.* trimester, *N/A* this CpG site is not annotated to any genes under Illumina UCSC annotation

* Position based on Human Genome Build 37 (hg19)

** Raw *p* values generated from linear regression analyses (DNA methylation regressed on case-control status and trimester-specific surrogate variables)

^ Sites carried forward for replication testing. Direction (cases vs. controls)

“+” indicates that DNA methylation values are higher in cases compared to controls while “—” indicates that DNA methylation values are lower in cases compared to controls

**Table 3:** Discovery phase differentially methylated regions with adjusted *p* values less than 0.05 for each trimester

Tri.	Chr.	Start position*	End position*	Gene name	Number of CpGs	Adjusted p value^^^

1	5	171710114	171710573	*UBTD2*	3	4.31 × 10^−3^
	10	27793008	27793165	*RAB18*	5	8.26 × 10^−7^
	15	75018774	75019302	*CYP1A1*	10	3.91 × 10^−3^
2	2	64067521	64067760	*UGP2*	3	3.28 × 10^−2^
	10	51572216	51572718	*NCOA4*	8	9.87 × 10^−4^
	19	59066476	59066632	*CHMP2A*	7	2.17 × 10^−2^
3	6	111902385	111902611	*TRAF3IP2-AS1; TRAF3IP2*	2**	5.53 × 10^−4^
	14	35761327	35761551	*PSMA6*	5	7.48 × 10^−3^
	17	80560593	80561084	*FOXK2*	4	1.27 × 10^−2^

Table ordered by trimester, chromosome, and position

*Chr.* chromosome, *Tri.* trimester

* Position based on Human Genome Build 37 (hg19)

^ Adjusted *p* values calculated as described in the “Methods and materials” section using the Bonferroni approach by dmrff

** Region consists of both cg218829900 and cg16155413 that were selected for trimester 3 replication

**Table 4:** Demographic and clinical characteristics of participants in the trimester 3 replication phase

Characteristics	Validation sample			Independent sample			Combined sample		
**Matched variables**	**Preeclampsia cases (*n*= 25)**	**Normotensive controls (*n*= 28)**	***p***	**Preeclampsia cases (*n*= 64)**	**Normotensive controls (*n*= 50)**	***p***	**Preeclampsia cases (*n*= 89)**	**Normotensive controls (*n*= 78)**	***p***
Race, *n* (%)									
*Black*	18 (72.0%)	21 (75.0%)	NA*	7 (10.9%)	5 (10.0%)	NA*	25 (28.1%)	26 (33.3%)	NA*
*White*	7 (28.0%)	7 (25.0%)		57 (89.1%)	45 (90.0%)		64 (71.9%)	52 (66.7%)	
Pre-pregnancy BMI, mean kg/m^2^ (SD)	32.5 (7.0)	33.7 (7.5)		25.5 (5.0)	24.7 (4.7)		27.5 (6.4)	28.0 (7.2)	
Gestational age at sample collection, mean weeks (SD)									
*Trimester 3*	37.0 (2.5)	37.4 (2.5)		36.7 (3.3)	38.1 (2.2)		36.8 (3.1)	37.9 (2.3)	
**Unmatched variables**	**Preeclampsia cases (*n*= 25)**	**Normotensive controls (*n*= 28)**	***p*** **	**Preeclampsia cases (*n*= 64)**	**Normotensive controls (*n*= 50)**	***p*** **	**Preeclampsia cases (*n*= 89)**	**Normotensive controls (*n*= 78)**	***p*** **
Parlty, *n* (%)									
*Nulliparous* (Yes)	18 (72.0%)	24 (85.7%)	0.10	52 (81.3%)	38 (76.0%)	0.48	70 (78.7%)	62 (79.5%)	0.89
(No)	7 (28.0%)	4 (14.3%)		12 (18.7%)	12 (24.0%)		19 (21.3%)	16 (20.5%)	
Maternal age at blrth, mean years (SD)	24.2 (5.2)	23.7 (4.2)	0.70	27.2 (6.7)	28.0 (5.8)	0.53	26.4 (6.5)	26.4 (5.7)	0.96
Gestational age at dellvery, mean weeks (SD)	37.3 (2.5)	39.5 (1.3)	6.16×10^−4^	36.8 (3.3)	38.8 (1.2)	8.46×10^−7^	36.9 (3.1)	39.1 (1.3)	5.09×10^−9^
Blood pressure <20 weeks, mean mmHg (SD)									
*Systoiic*	113.0 (7.7)	111.5 (8.9)	0.40	114.2 (9.4)	114.7 (8.4)	0.78	113.9 (8.9)	113.5 (8.7)	0.80
*Missing*	0	0	/	3	1	/	3	1	/
*Diastolic*	70.0 (5.9)	66.8 (6.1)	0.05	70.2 (7.4)	69.7 (5.3)	0.71	70.1 (7.0)	68.7 (5.7)	0.15
*Missing*	0	0	/	3	1	/	3	1	/
Blood pressure prior to dellvery, mean mmHg (SD)									
*Systoiic*	148.8 (7.1)	124.5 (6.1)	1.02×10^−2^	155.5 (15.1)	120.8 (11.9)	2.43×10^−19^	153.6 (13.7)	122.1 (10.3)	9.82×10^−28^
*Diastolic*	91.2 (8.3)	71.1 (6.2)	3.54×10^−9^	92.6 (8.8)	73.8 (7.3)	5.83×10^−18^	92.2 (8.6)	72.8 (7.0)	3.30×10^−26^

Validation sample, all trimester 3 samples from the discovery phase were sent for validation with the pyrosequencing platform; Independent sample, trimester 3 samples from test sample completely independent of the discovery phase; Combined sample, pooled validation and independent samples

* Variables matched based on self-identified race, pre-pregnancy BMI, and gestational age at sample collection (± 2 weeks when possible) so *p* values are not reported

** *P* values from the partially overlapping samples *t* test for continuous variables and partially overlapping samples *z* test for dichotomous variable

**Table 5:** Replication phase results examining trimester 3 DNAm data in the discovery (i.e., validation), replication, and combined samples

Replication phase	N/n_control_/n_case_	N_pairs_*	Mean DNA methylation as beta-values (%)		Mean DNA methylation as M-values		Direction (Cases vs. Controls)	*p* **
			Controls	Cases	Controls	Cases		

**chr6:111902626**								
Validation sample	53/28/25	25	72.24	78.93	1.41	1.94	+	9.53×10^−5^
Independent sample	112/50/62	43	75.16	73.14	1.67	1.49	−	0.11
Combined sample	165/78/87	68	74.11	74.81	1.58	1.62	+	0.65
**cg16155413, chr6:111902611**								
Validation sample	51/28/23	23	46.20	56.34	−0.22	0.37	+	1.05×10^−4^
Independent sample	93/38/55	27	53.04	48.73	0.18	−0.08	−	0.06
Combined sample	144/66/78	50	50.13	50.98	0.007	0.05	+	0.68
**cg21882990, chr6:111902385**								
Validation sample	50/26/24	22	81.10	82.05	2.11	2.20	+	0.04
Independent sample	77/30/47	25	80.63	79.47	2.08	1.99	−	0.27
Combined sample	127/56/71	47	80.85	80.34	2.09	2.06	−	0.54

Validation sample, all trimester 3 samples from the discovery phase were sent for validation with the pyrosequencing platform; Independent sample, trimester 3 samples from test sample completely independent of the discovery phase; Combined sample, pooled validation and independent samples

* Number of matched pairs for which both observations were present

** *p* values from the partially overlapping samples *t* test of *M* values

## Data Availability

The dataset supporting the conclusions of this article is available in dbGAP, accession number: phs001937.v1.p1.

## Abbreviations

PE: Preeclampsia
DNAm: DNA methylation
EWAS: Epigenome-wide association analysis
SVA: Surrogate variable analysis
DMR: Differentially methylated region
BMI: Body mass index
QQ plot: Quantile-quantile plot
SNP: Single-nucleotide polymorphism
SD: Standard deviation
QC: Quality control
