# Supplementary material for: A longitudinal epigenome-wide association study of preeclamptic and normotensive pregnancy
Source: Epigenetics Commun. Author manuscript; Available in PMC 2023 Apr 13. (PMC10101051; doi:10.1186/s43682-022-00014-w)
Supplement: AdditionalFile1 [file NIHMS1884910-supplement-AdditionalFile1.pdf]

Supplementary Materials

for

A longitudinal epigenome-wide association study of preeclamptic and normotensive pregnancy

Shuwei Liu^, Haoyi Fu^, Mitali Ray, Lacey W. Heinsberg, Yvette P. Conley, Cindy M. Anderson, Carl A. Hubel, James M. Roberts, Arun Jeyabalan, Daniel E. Weeks, Mandy J. Schmella*

^*Equal contributions as first-authors*
**Corresponding Author,* mjb111@pitt.edu

Quality Control

Discovery phase sample availability

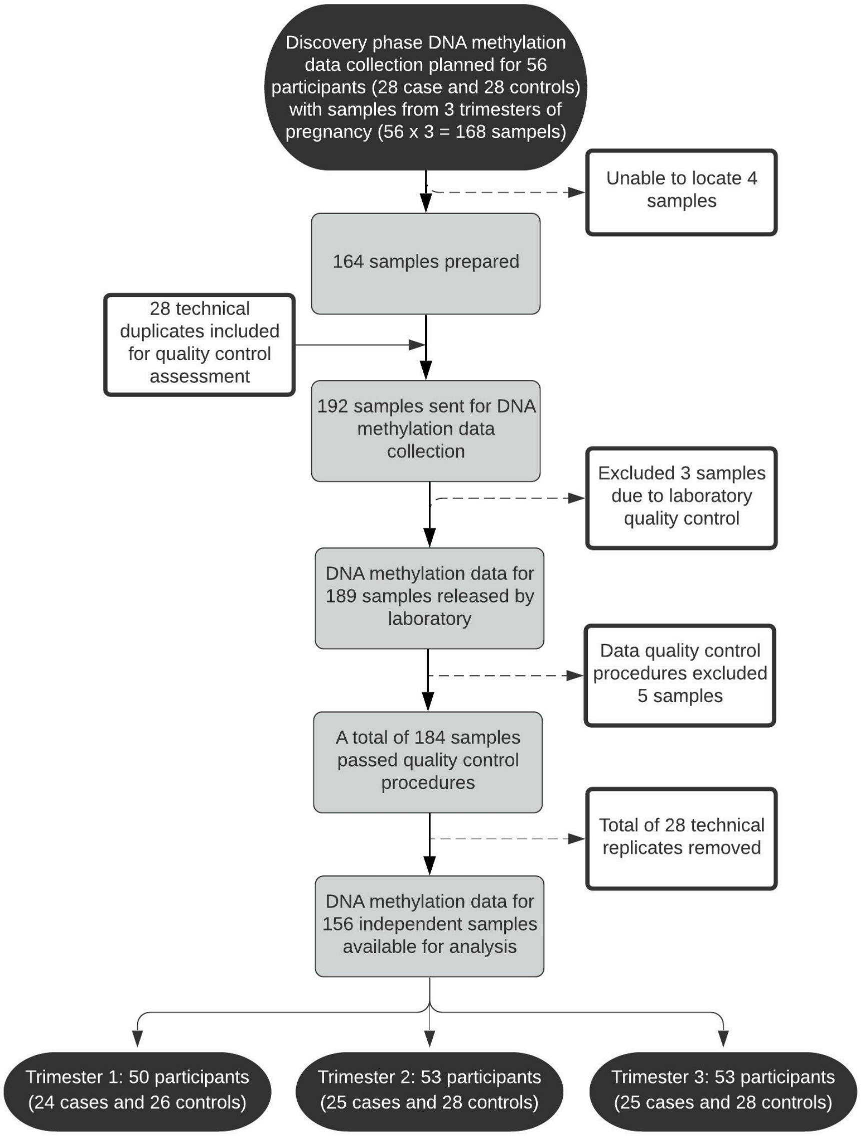

Figure S1. Overview of discovery phase data collection and quality control

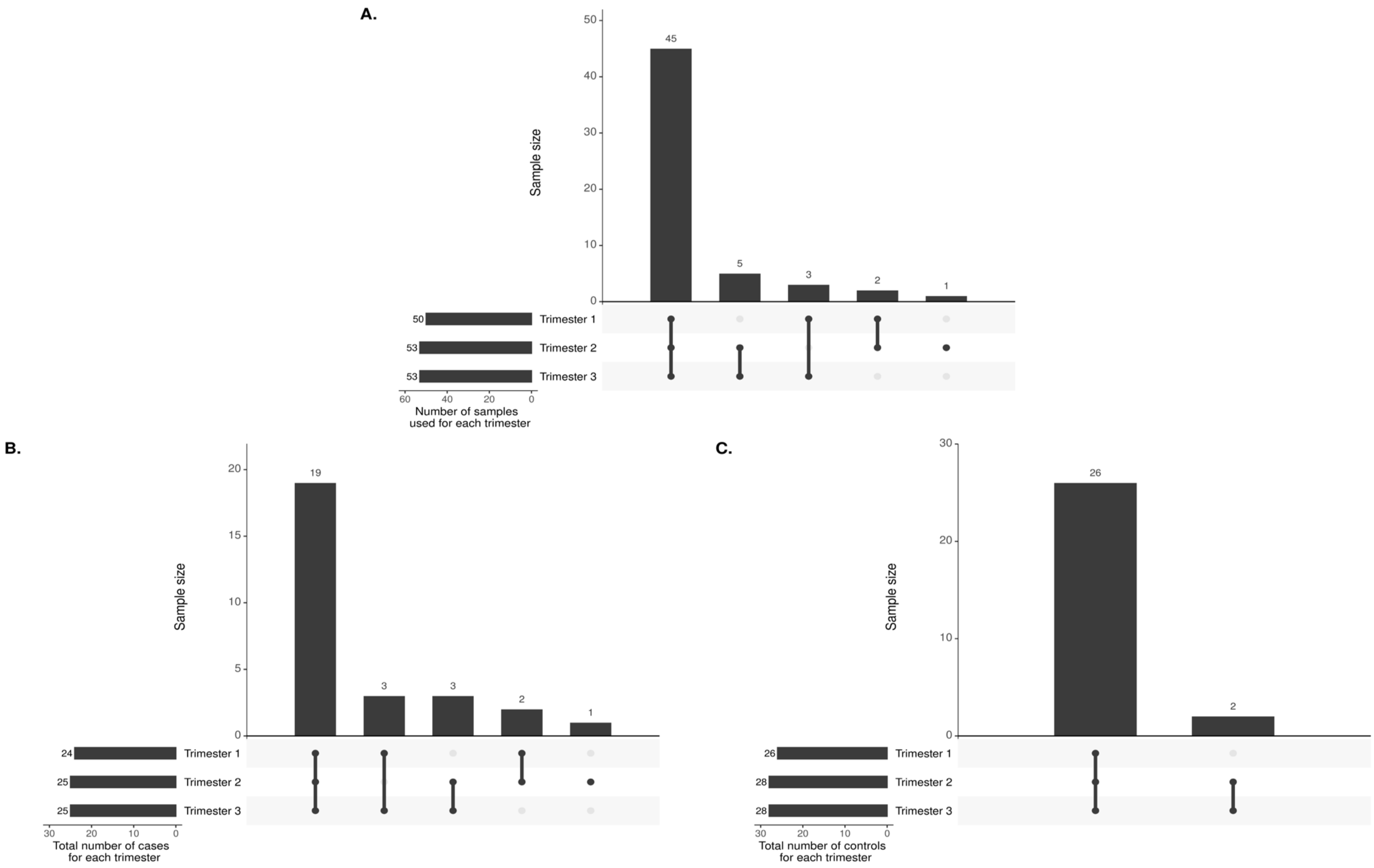

Figure S2. Intersection plots of samples included in each trimester.
A. All samples, **B.** Cases only, **C.** Controls only

Quality control report and issues

We used the R minfi and ENmix packages to examine the density plot of β values, mean detection of p-values for each sample, averaged bisulfite conversion intensity plot, and performed data quality control (1–4). CpG probes are multimodal and have two peaks as shown in the β value density plot (**Figure S3**).

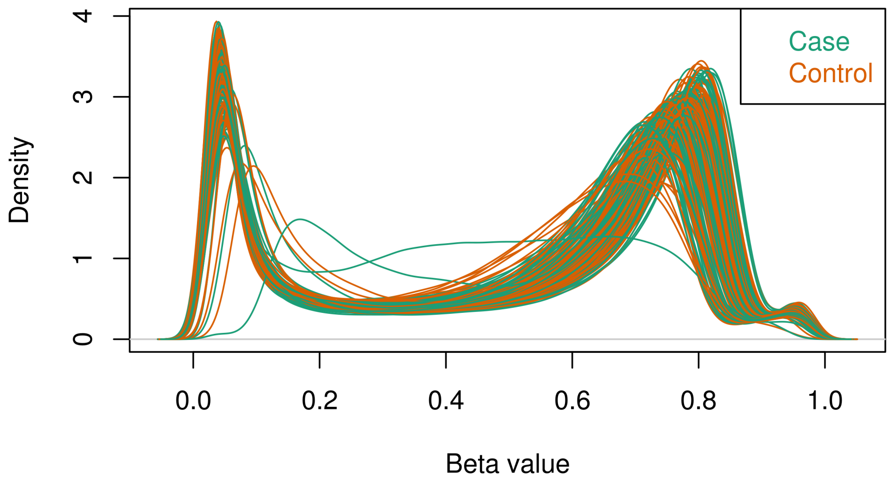

Figure S3. Beta density plot for raw data

Most samples had low mean detection p-values, with only one sample having mean detection p-values larger than 0.01 (**Figure S4**).

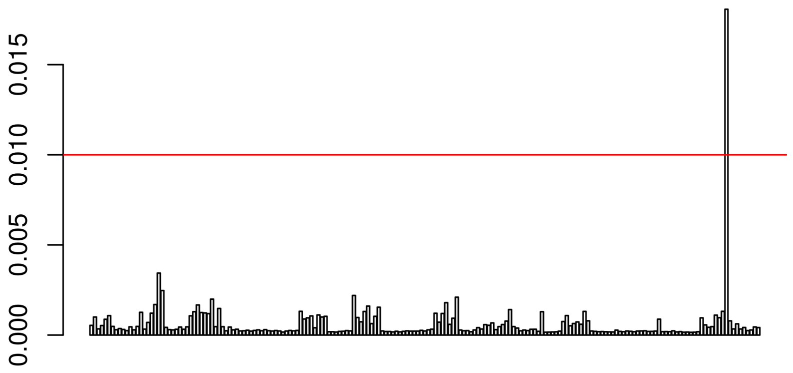

Figure S4. Mean detection p-values for each sample

In examining that sample in more detail, it was identified as “bad” since it did not cluster with other samples based on median intensities (**Figure S5**).

In total, 5 samples were identified as “bad” samples in Enmix quality control checks where 4 samples were low quality samples with the percentage of low quality CpGs greater than 0.01, and 1 sample was an outlier in the beta value distribution.

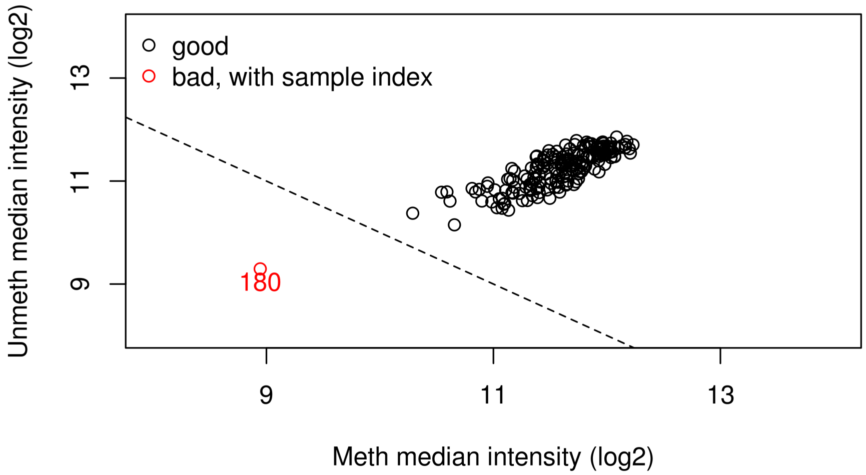

Figure S5. Clustering of samples based on median intensities

Next, DNA methylation levels between technical replicates were checked. In the raw data, discrepancies between methylation levels of some technical replicates indicated potential sources of unwanted variation (**Figure S6** and **S7**).

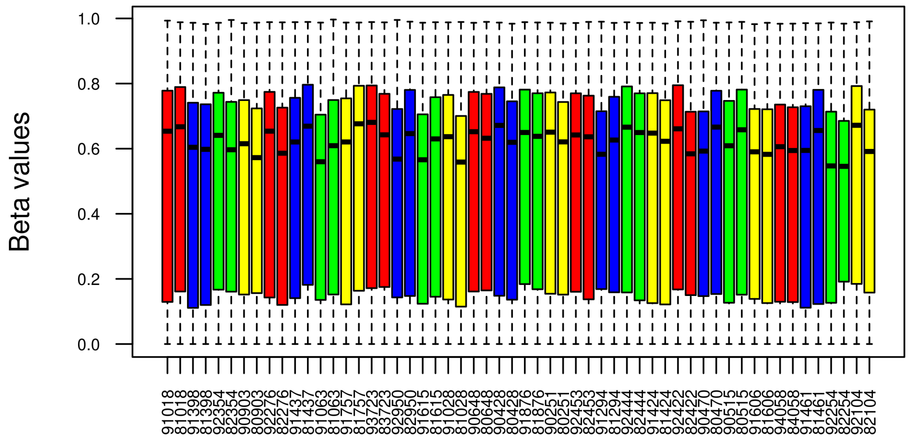

Figure S6. Raw methylation beta values of technical replicates before normalization
Adjacent samples of the same color are replicates. The black horizontal line is the mean beta value for each sample.

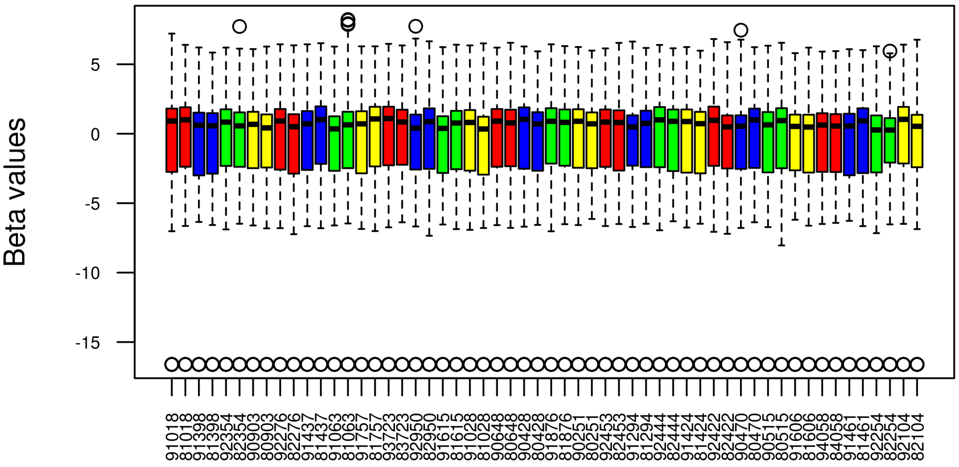

Figure S7. Raw methylation M values of technical replicates before normalization
Adjacent samples of the same color are replicates. The black horizontal line is the mean beta value for each sample.

Next we used the R lumi package to take a more detailed look at the relationship between technical replicates using Multi-Dimensional Scaling (MDS) or hierarchical clustering methods[23] using clustering plots (5). Based on our laboratory design and the inclusion of 28 technical replicates, we expected 28 "true pairs" (i.e., perfect matches). In looking at the raw data, however, the initial clustering prior to normalization did not align well. Out of the 28 duplicate pairs, there were only 15 true pairs identified (**Figure S8**).

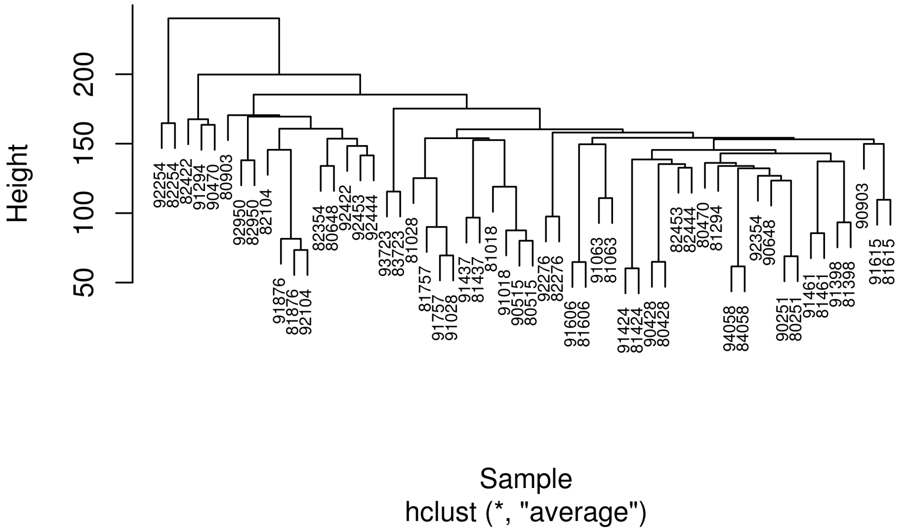

Figure S8. Clustering for all duplicates before normalization
A "true" or "correct" pairing occurs when the last four digits of the sample ID match.

Normalization
Next, functional normalization was performed using the funtooNorm package which was designed to not only perform this critical quality control step, but also adjust for multiple cell types and tissues (6). This approach is an extension of the functional normalization method available in the minfi package (1).

The funtooNorm package allows the flexibility to select the ideal number of components using cross-validation, which are computed based on the control probes and cell type data. Principal component regression (PCR) or partial least square regression (PLS) model is fit for the funtooNorm normalization (6). Our DNA methylation data were unique in that they were longitudinal across three trimesters of pregnancy. Thus, we treated trimester as the "cell type" variable to retain variation due to time.

This package has a function which can be used to choose the number of components based on the cross-validation for each methylation channel. Based on our results, setting the number of components equal to 3 was an ideal choice as it produced a significantly lower RMSE compared to setting the number of components equal to 1 or 2 (**Figure S9**).

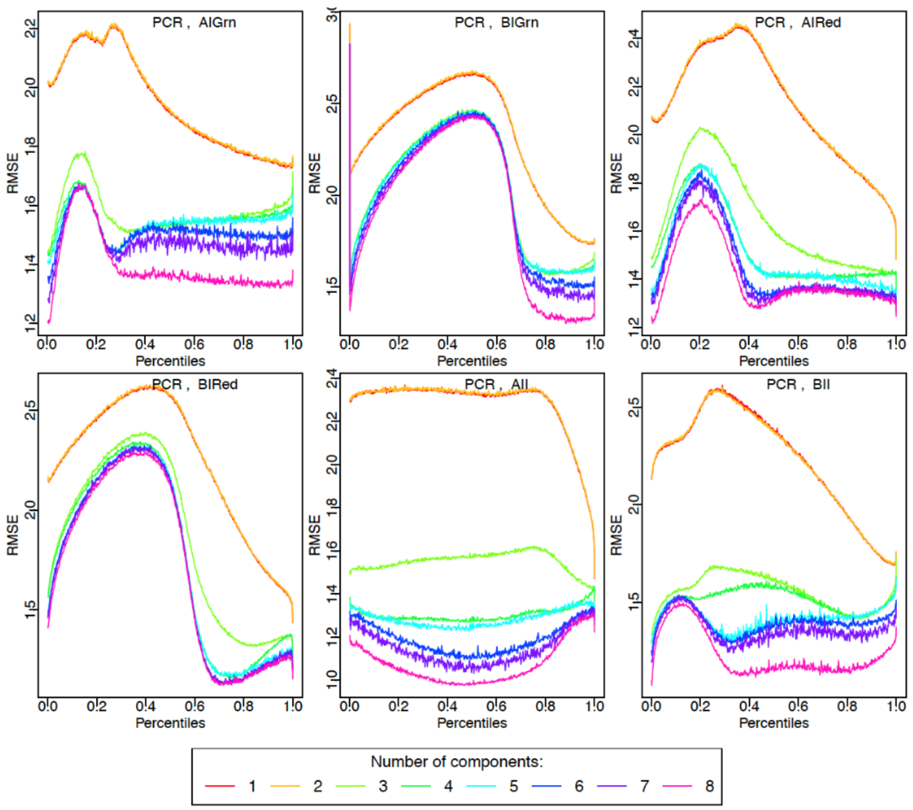

Figure S9. Selection of number of components based on 10-fold cross-validation

Removal of bad probes

In terms of CpG probes, raw DNA methylation data were returned for 865,859 CpGs before quality control procedures by minfi. Our quality control procedures included (1) removal of probes with SNPs at CpG or single base extension (SBE) sites; (2) removal of cross-reactive probes; (3) since our study only included females, CpG probes on the Y chromosome were removed; and (4) removal of "bad" probes identified by ENmix (percentage of low quality CpG sites across samples greater than 0.05 whose detection p-values were greater than 0.01 or number of beads less than 3). Table S1 shows number of CpGs removed by each quality control step.

The density plot of normalized data after quality control is shown in Figure S10.

Table S1. Number of CpGs removed by quality control steps

Removed step	Number of CpGs	Number of removed CpGs
**Raw**	865859	NA
**Probes with SNPs**	835424	30435
**Cross-reactive probes**	708473	126951
**Y chromosome probes**	708094	379
**ENmix bad probes***	703200	4894

* Enmix bad probes refer to any remaining bad probes that were identified by R Enmix package. The "bad probes" was identified if the percentage of a CpG site across samples having detection p-values greater than 0.01 was greater than 0.05 or the number of beads less than 3 .

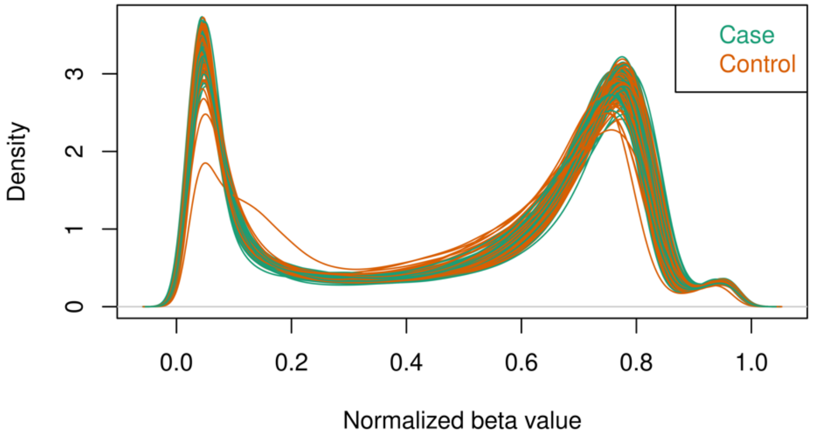

Figure S10. Post-quality control beta density plot

Clustering analysis of duplicate pairs
Next, duplicate pairs were again examined to assess overall performance of quality control procedures. In repeating the examination of clustering for all technical replicates across all trimesters, 18 out of 28 pairs were true pairs. Thus, the funtooNorm method improved the clustering results compared to the raw data clustering results.

To examine the clustering of samples and replicates across each trimester, we created clustering and bar plots to compare each sample and its replicate within each trimester. Perfect matching (9 out of 9 duplicate pairs) was displayed for pregnancy trimester 1 (**Figure S11**). For trimester 2, 6 out of 10 were true pairs (**Figure S13**) and for trimester 3, 8 out of 9 were true pairs (**Figure S15**). These results demonstrated that funtooNorm successfully reduced unwanted sources of variation.

Boxplots depicting the methylation levels between technical replicates support this as most duplicate pairs had the same or very similar levels of methylation within each trimester (**Figure S12, S14,** and **S16**).

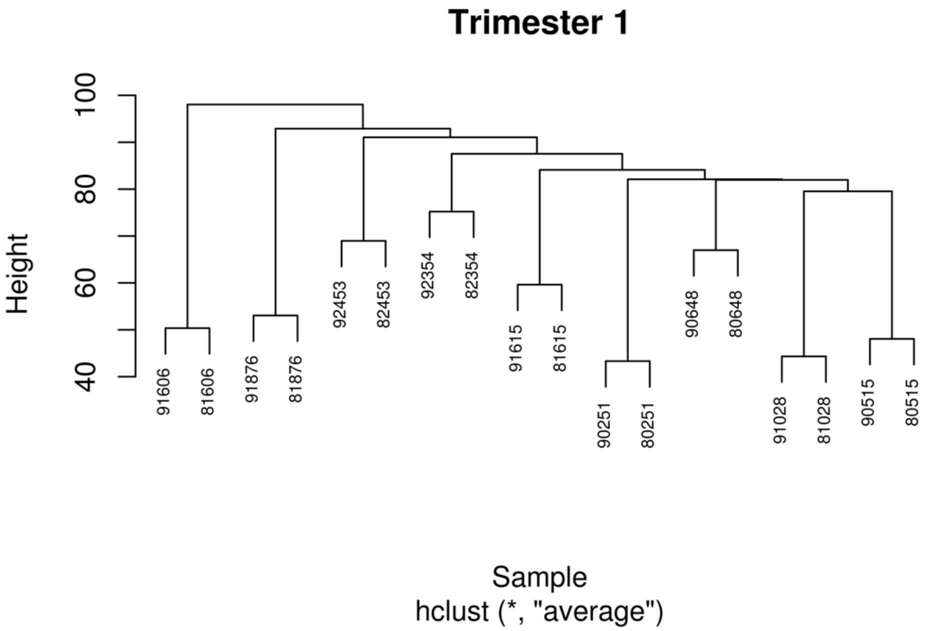

Figure S11. Post-quality control clustering plot for trimester 1 based on beta values
A "true" or "correct" pairing occurs when the last four digits of the sample ID match.

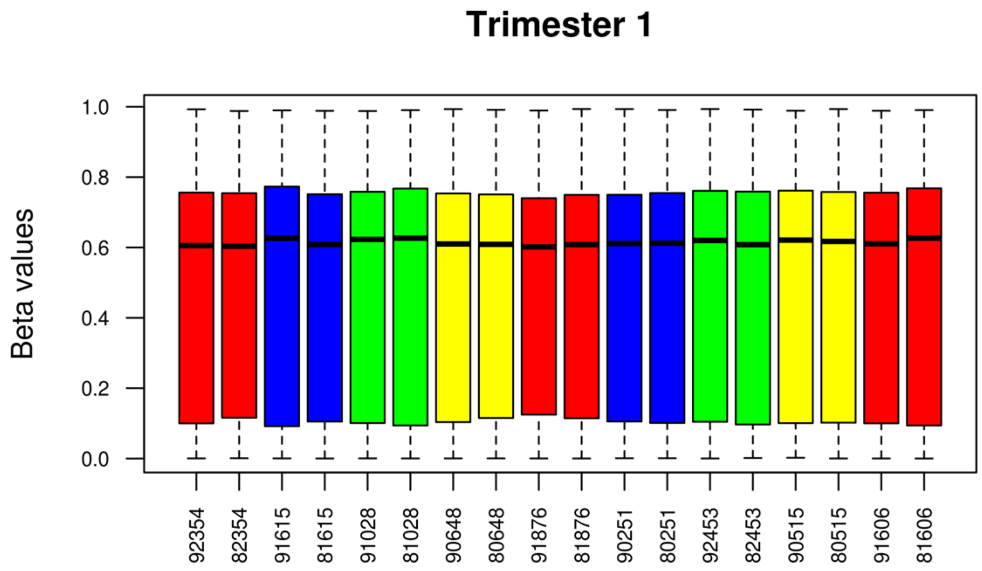

Figure S12. Post-quality control beta values for duplicates in trimester 1

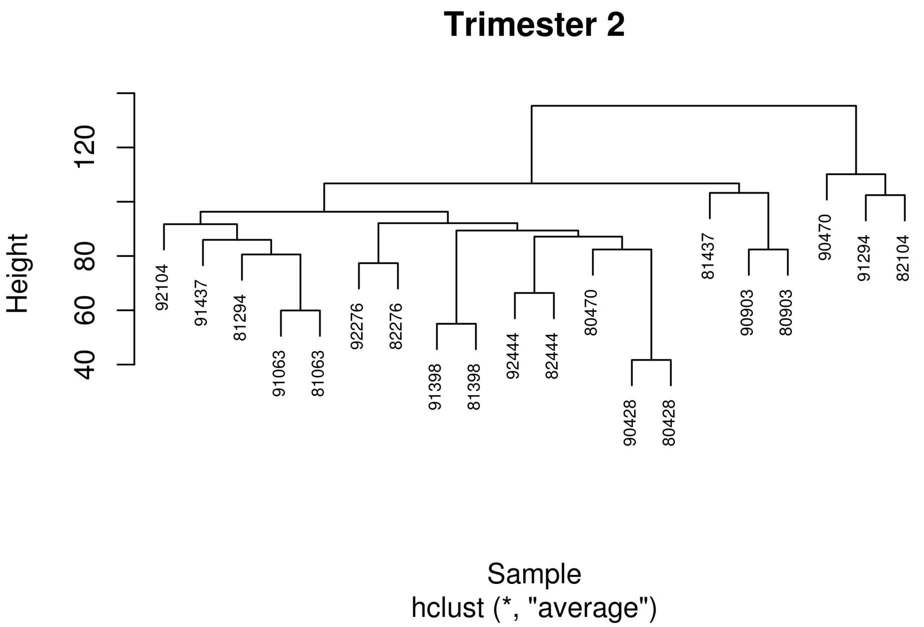

Figure S13. Post-quality control clustering plot for trimester 2 based on beta values

A "true" or "correct" pairing occurs when the last four digits of the sample ID match.

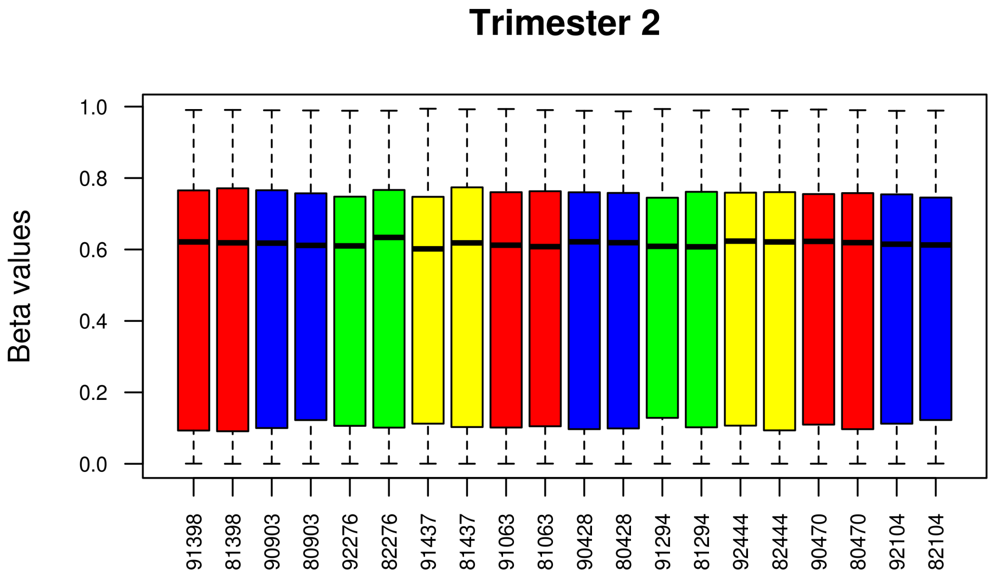

Figure S14. Post-quality control beta values for duplicates in trimester 2

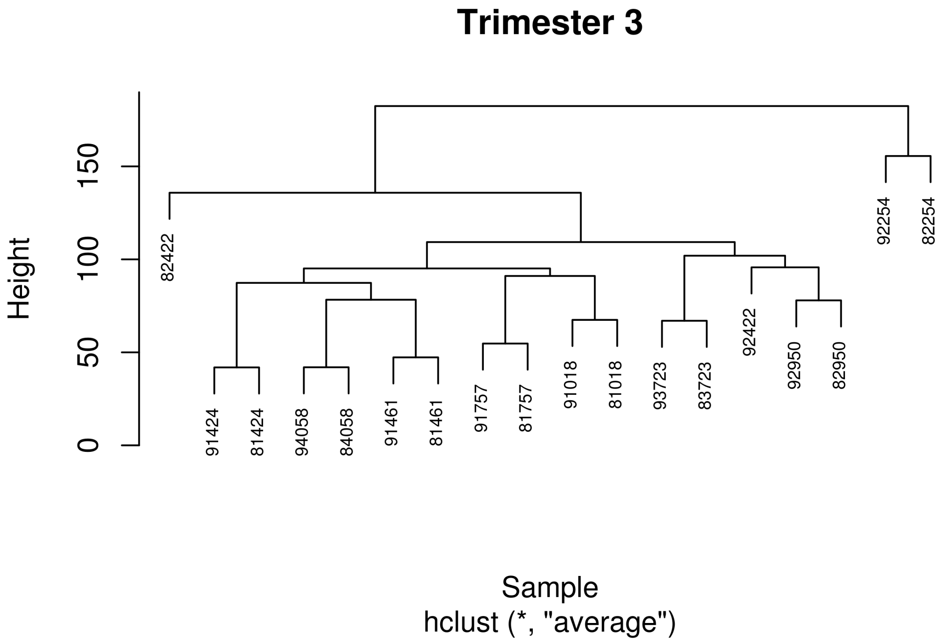

Figure S15. Post-quality control clustering plot for trimester 3 based on beta values

A “true” or “correct” pairing occurs when the last four digits of the sample ID match.

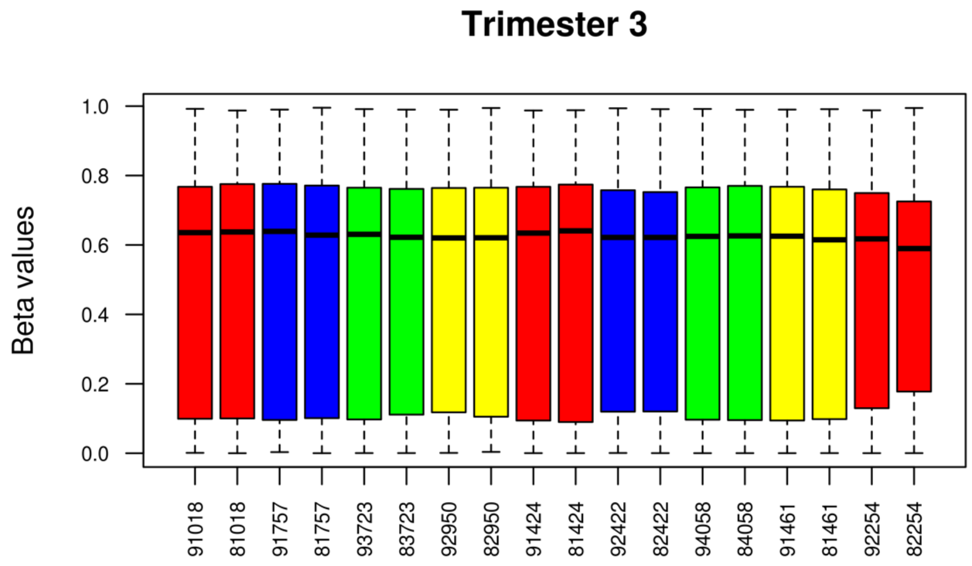

Figure S16. Post-quality control beta values for duplicates in trimester 3

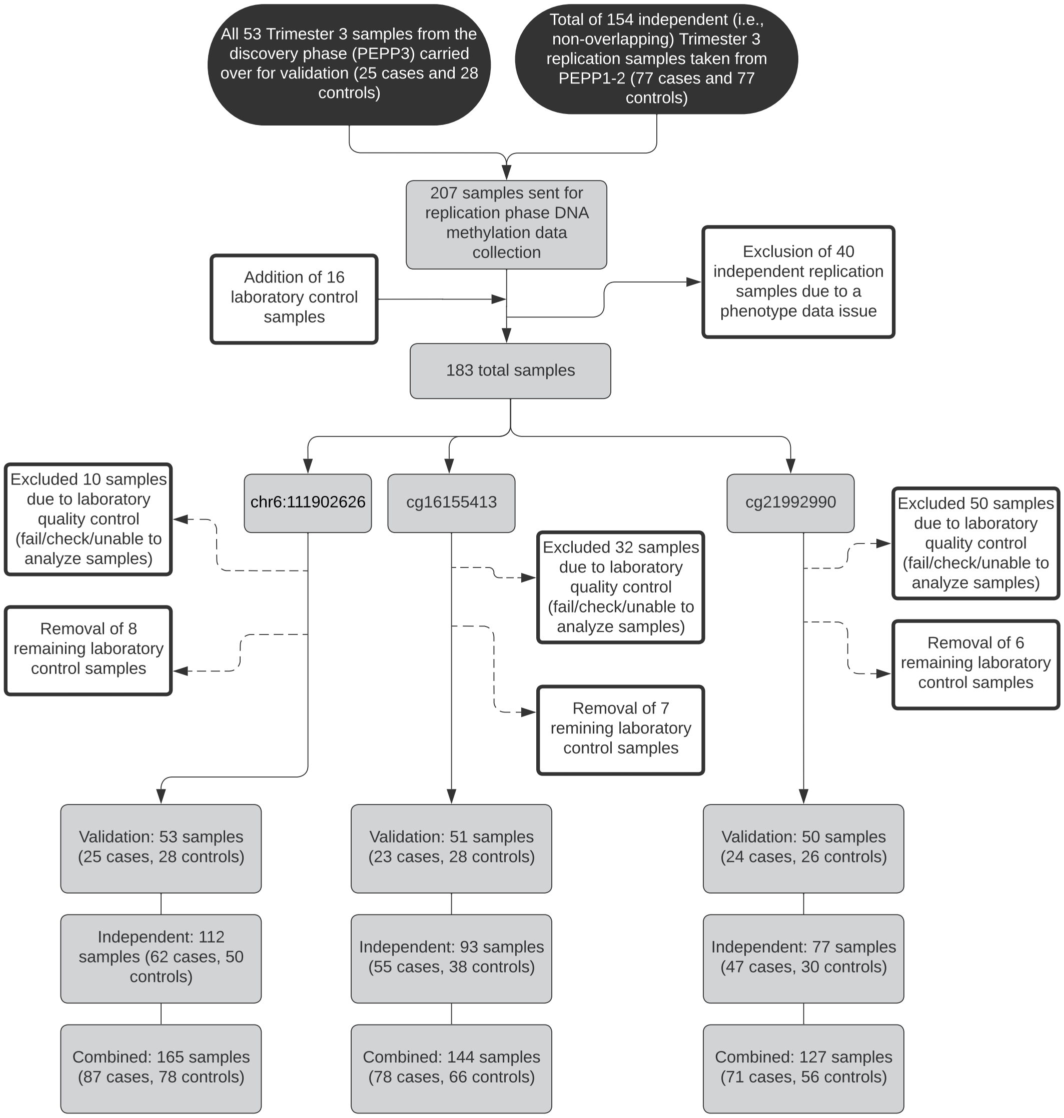

Figure S17. Overview of replication phase data collection and quality control

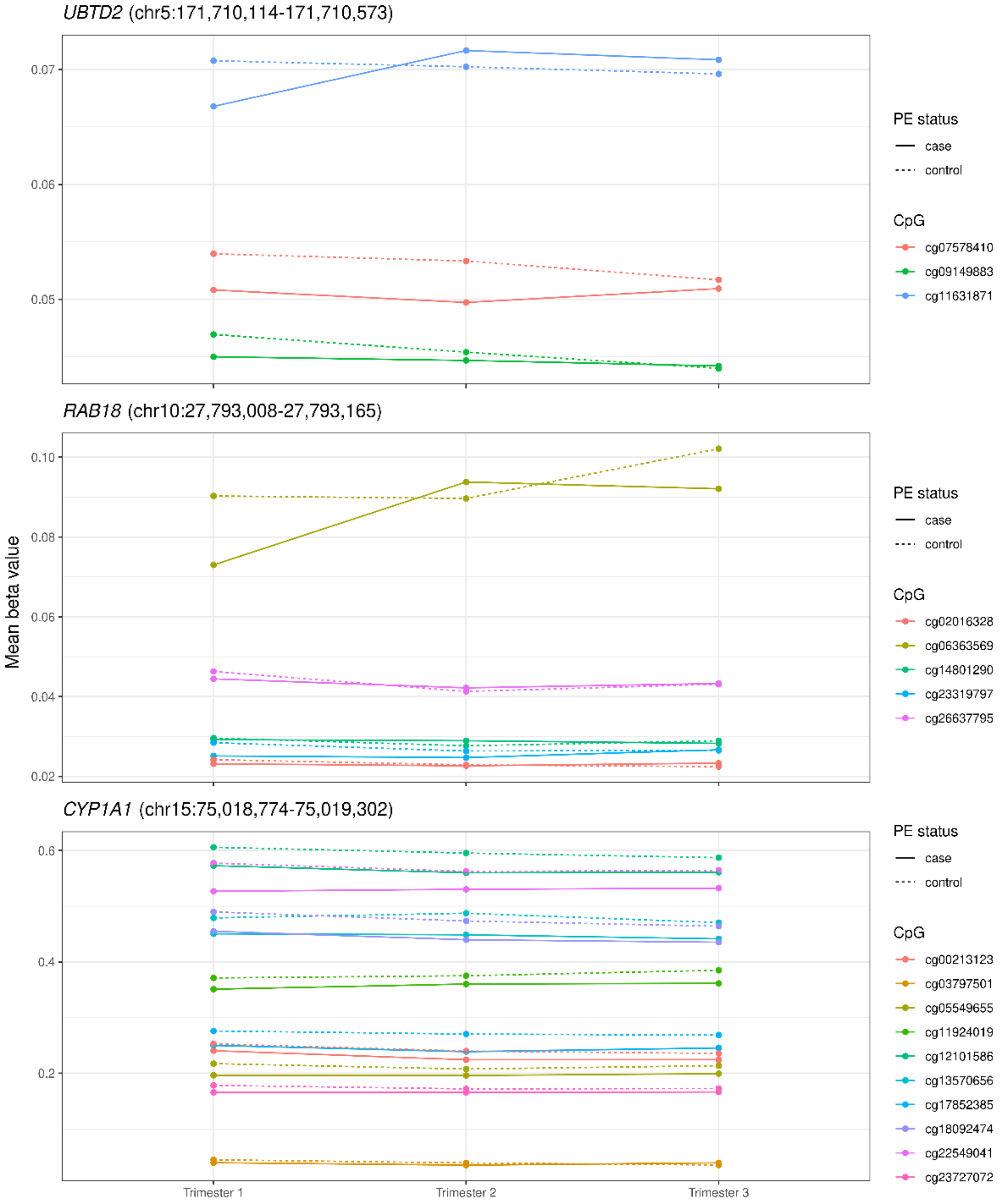

Figure S18. Trajectory plot of mean DNAm beta values from each DMRs in discovery phase across three trimesters

This plot shows the trajectories of the three significant DMRs identified in Trimester 1.

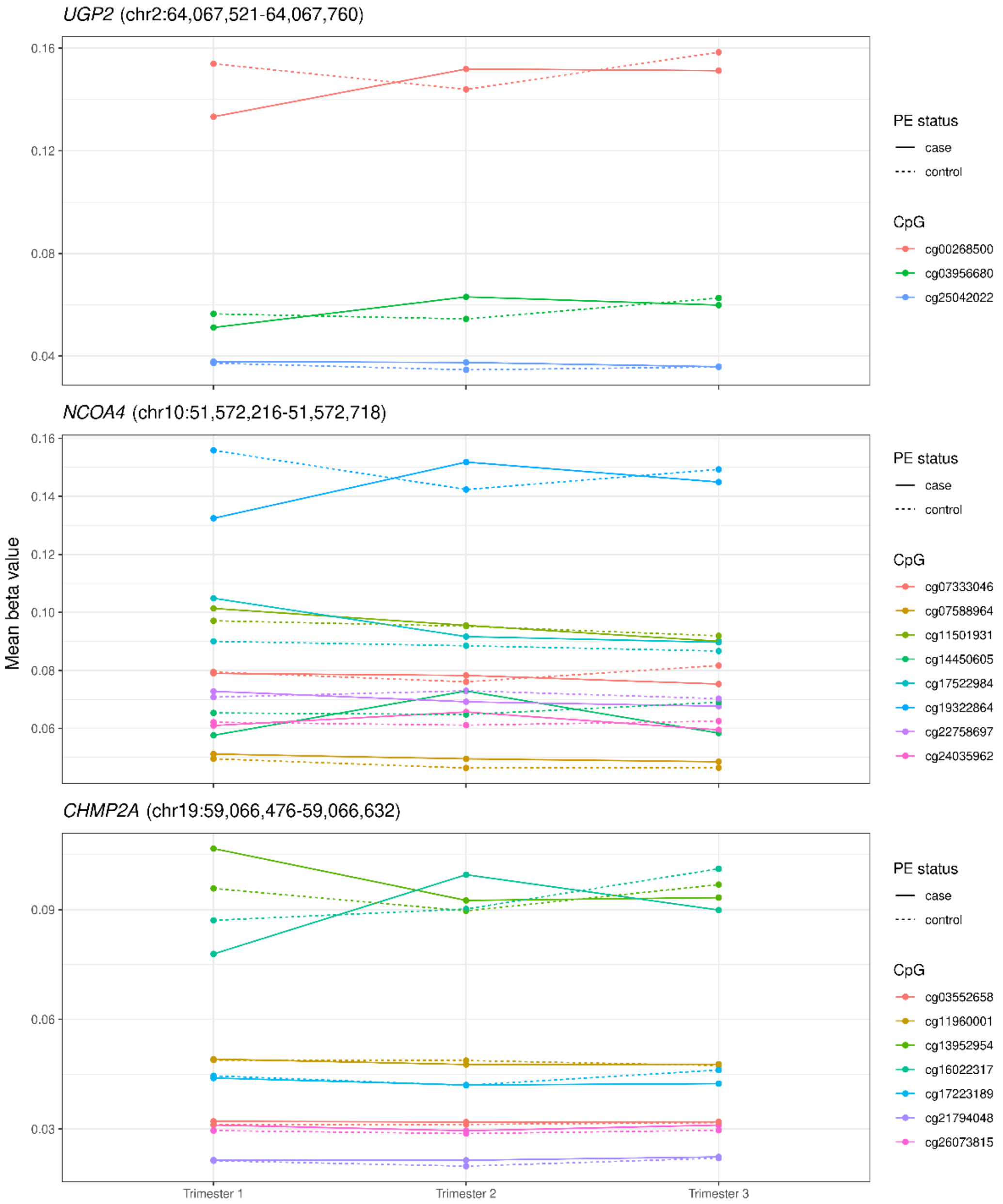

Figure S19. Trajectory plot of mean DNAm beta values from each DMRs in discovery phase across three trimesters

This plot shows the trajectories of the three significant DMRs identified in Trimester 2.

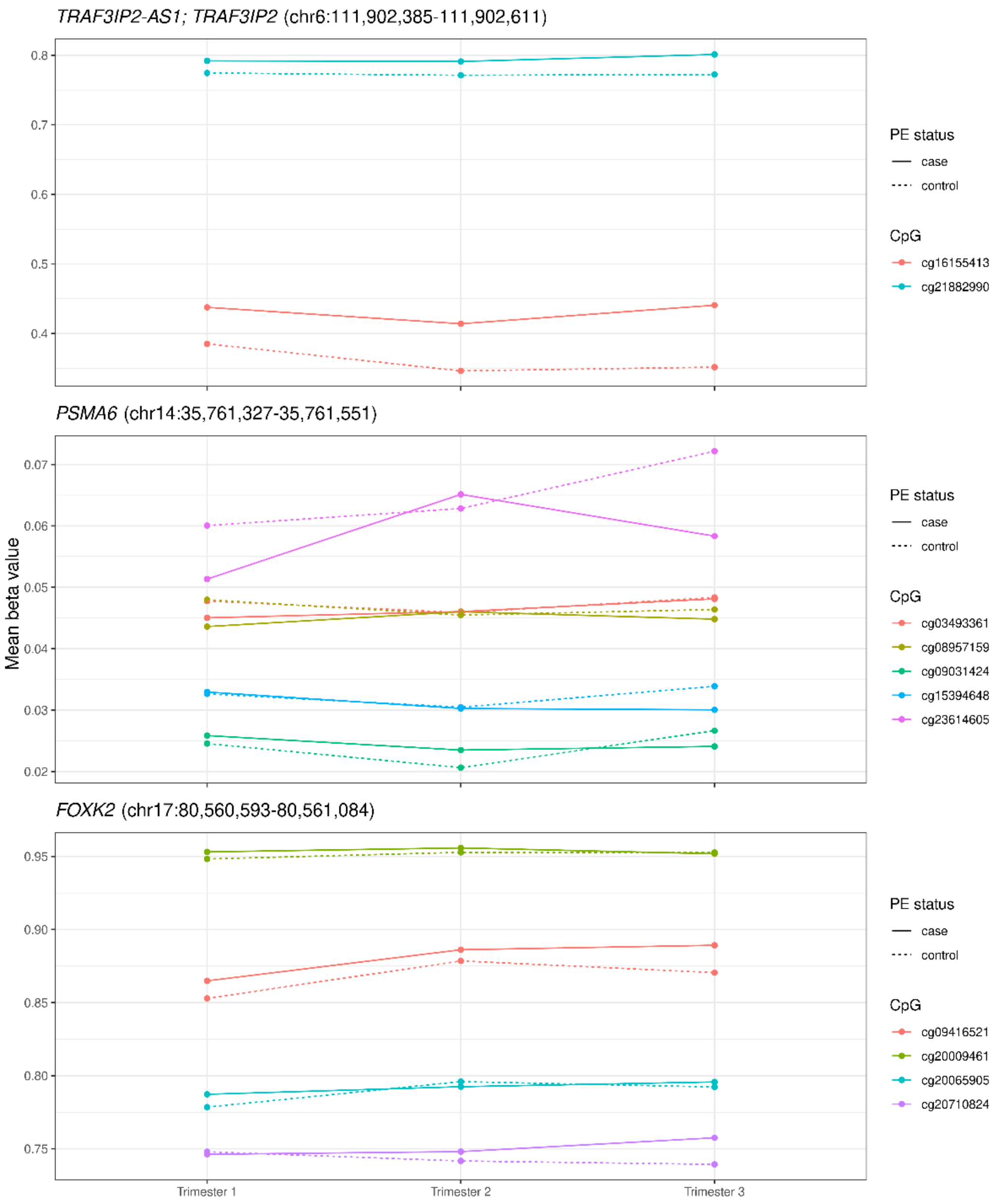

Figure S20. Trajectory plot of mean DNAm beta values from each DMRs in discovery phase across three trimesters

This plot shows the trajectories of the three significant DMRs identified in Trimester 3.

Table S2. Pyrosequencing assay information for replication samples

Gene	*TRAF3IP2-AS1; TRAF3IP2*	*TRAF3IP2-AS1; TRAF3IP2*
Illumina Probe	cg16155413	cg21882990
PyroMark CpG Assay	Hs_CG16155413_01_PM	Hs_CG21882990_02_PM
Geneglobe Catalog Number	PM00681443	PM00681415
Sequence before bisulfite treatment	TTACGCCTCCTCCTGGATCGTCTTAC	ACCTAATGCTTAATCACGC
Sequence to analyze	TTAYGTTTTTTTTTGGATYGTTTTAT	ATTTAATGTTTAATTAYGT
Total number of sites	2	1
Total number of sites examined (i.e., passed QC)	2	1

Table S3. Summary of replication data QC

	Position[a]	Pass	Check	Fail	NA	Total[b]	% Fail
TRAF3IP2-AS1; TRAF3IP2 - cg16155413							
Site 1	chr6: 111902626	173	2	0	8	183	0%
Site 2 (cg16155413)	chr6: 111902611	151	18	6	8	183	3.3%
TRAF3IP2-AS1; TRAF3IP2 - cg21882990							
Site 1 (cg21882990)	chr6: 111902385	133	42	3	5	183	1.6%

[a] Position based on Human Genome Build 37 (hg19); [b] Total number includes laboratory controls.

Table S4. Associations of DNAm data and PE status in the discovery (i.e., validation), replication, and combined samples of replication phase with adjustment for race, pre-pregnancy BMI, and maternal age at birth.

Part A: Linear regression analyses

Variables[a]	chr6:111902626											
	Validation sample (n = 53)				Independent sample (n = 112)				Combined sample (n = 165)			
	$\hat{\beta}$	SE[b]	95%CI[c]	*p* value	$\hat{\beta}$	SE	95%CI	*p* value	$\hat{\beta}$	SE	95%CI	*p* value
Intercept	2.22				1.83				1.82			
PE status (reference: controls)	0.50	0.12	[0.26, 0.74]	1.35×10^{-4}	−0.18	0.12	[−0.42, 0.06]	0.13	0.04	0.09	[−0.15, 0.22]	0.70
Race (reference: Black)	0.30	0.17	[−0.03, 0.64]	0.08	0.10	0.20	[−0.29, 0.48]	0.63	0.03	0.11	[−0.20, 0.25]	0.82
Pre-pregnancy BMI	−0.02	0.009	[−0.04, −0.001]	0.04	-3.96×10^{-3}	0.01	[−0.03, 0.02]	0.75	-4.58×10^{-3}	0.007	[−0.02, 0.009]	0.52
Maternal age at birth	−0.01	0.02	[−0.04, 0.02]	0.51	-5.10×10^{-3}	0.01	[−0.02, 0.01]	0.60	-5.10×10^{-3}	0.008	[−0.02, 0.01]	0.55
	cg16155413, chr6:111902611											
	Validation sample (n = 51)				Independent sample (n = 93)				Combined sample (n = 144)			
	$\hat{\beta}$	SE	95%CI	*p* value	$\hat{\beta}$	SE	95%CI	*p* value	$\hat{\beta}$	SE	95%CI	*p* value
Intercept	0.46				0.77				0.35			
PE status (reference: controls)	0.57	0.12	[0.33, 0.82]	2.50×10^{-5}	−0.26	0.14	[−0.54, 0.02]	0.07	0.04	0.11	[−0.17, 0.25]	0.70
Race (reference: Black)	0.25	0.17	[−0.09, 0.60]	0.14	−0.37	0.23	[−0.82, 0.08]	0.10	−0.12	0.13	[−0.37, 0.13]	0.34
Pre-pregnancy BMI	−0.01	0.009	[−0.03, 0.003]	0.10	−0.01	0.01	[−0.04, 0.02]	0.38	−0.01	0.008	[−0.03, 0.004]	0.15
Maternal age at birth	−0.01	0.02	[−0.04, 0.02]	0.51	1.92×10^{-3}	0.01	[−0.02, 0.03]	0.87	2.19×10^{-3}	0.01	[−0.02, 0.02]	0.82
	cg21882990, chr6:111902385											
	Validation sample (n = 50)				Independent sample (n = 77)				Combined sample (n = 127)			
	$\hat{\beta}$	SE	95%CI	*p* value	$\hat{\beta}$	SE	95%CI	*p* value	$\hat{\beta}$	SE	95%CI	*p* value
Intercept	1.95				1.47				1.75			
PE status (reference: controls)	0.08	0.04	[0.002, 0.17]	0.05	−0.08	0.09	[−0.26, 0.11]	0.40	−0.02	0.06	[−0.14, 0.09]	0.67
Race (reference: Black)	0.04	0.06	[−0.08, 0.15]	0.54	0.16	0.15	[−0.15, 0.46]	0.31	6.06×10^{-3}	0.07	[−0.13, 0.15]	0.93
Pre-pregnancy BMI	-9.89×10^{-4}	0.003	[−0.007, 0.005]	0.75	0.01	0.01	[−0.005, 0.03]	0.15	9.61×10^{-3}	0.005	[0.0005, 0.02]	0.04
Maternal age at birth	7.67×10^{-3}	0.005	[−0.003, 0.02]	0.16	2.61×10^{-3}	0.008	[−0.01, 0.02]	0.74	1.67×10^{-3}	0.006	[−0.009, 0.01]	0.77

[a] Linear regression model: DNAm M value ~ PE status + Race + Pre-pregnancy BMI + Maternal age at birth. [b] SE: standard error. [c] 95%CI: 95% confidence interval.
This table shows the association results including only samples with a "Pass" laboratory designation.

Part B: Cluster-robust standard errors analyses

Variables[a]	chr6:111902626											
	Validation sample (n = 53)				Independent sample (n = 112)				Combined sample (n = 165)			
	$\hat{\beta}$	SE[b]	95%CI[c]	*p* value	$\hat{\beta}$	SE	95%CI	*p* value	$\hat{\beta}$	SE	95%CI	*p* value
Intercept	2.22				1.83				1.82			
PE status (reference: controls)	0.50	0.12	[0.26, 0.74]	1.43×10^{-4}	−0.18	0.11	[−0.40, 0.04]	0.11	0.04	0.09	[−0.14, 0.22]	0.69
Race (reference: Black)	0.30	0.16	[−0.01, 0.63]	0.06	0.10	0.21	[−0.31, 0.50]	0.64	0.03	0.11	[−0.19, 0.24]	0.80
Pre-pregnancy BMI	−0.02	0.007	[−0.03, −0.005]	0.01	-4.00×10^{-3}	0.01	[−0.03, 0.02]	0.74	-4.60×10^{-3}	0.006	[−0.02, 0.007]	0.44
Maternal age at birth	−0.01	0.01	[−0.04, 0.02]	0.46	-5.10×10^{-3}	0.01	[−0.02, 0.01]	0.59	-5.10×10^{-3}	0.009	[−0.02, 0.01]	0.55
	**cg16155413, chr6:111902611**											
	Validation sample (n = 51)				Independent sample (n = 93)				Combined sample (n = 144)			
	$\hat{\beta}$	SE	95%CI	*p* value	$\hat{\beta}$	SE	95%CI	*p* value	$\hat{\beta}$	SE	95%CI	*p* value
Intercept	0.46				0.77				0.35			
PE status (reference: controls)	0.57	0.13	[0.30, 0.85]	9.86×10^{-5}	−0.26	0.14	[−0.54, 0.02]	0.07	0.04	0.11	[−0.18, 0.26]	0.71
Race (reference: Black)	0.25	0.15	[−0.04, 0.55]	0.09	−0.37	0.24	[−0.86, 0.11]	0.13	−0.12	0.12	[−0.35, 0.11]	0.31
Pre-pregnancy BMI	−0.02	0.006	[−0.03, −0.004]	9.23×10^{-3}	−0.01	0.02	[−0.04, 0.02]	0.42	−0.01	0.007	[−0.03, 0.002]	0.10
Maternal age at birth	−0.01	0.02	[−0.04, 0.02]	0.52	1.92×10^{-3}	0.01	[−0.02, 0.03]	0.87	2.19×10^{-3}	0.01	[−0.02, 0.02]	0.84
	**cg21882990, chr6:111902385**											
	Validation sample (n = 50)				Independent sample (n = 77)				Combined sample (n = 127)			
	$\hat{\beta}$	SE	95%CI	*p* value	$\hat{\beta}$	SE	95%CI	*p* value	$\hat{\beta}$	SE	95%CI	*p* value
Intercept	1.95				1.47				1.75			
PE status (reference: controls)	0.08	0.04	[0.005, 0.17]	0.04	−0.08	0.08	[−0.25, 0.09]	0.35	−0.02	0.06	[−0.14, 0.09]	0.66
Race (reference: Black)	0.04	0.06	[−0.09, 0.16]	0.57	0.16	0.11	[−0.07, 0.38]	0.16	6.06×10^{-3}	0.08	[−0.16, 0.17]	0.94
Pre-pregnancy BMI	-9.89×10^{-4}	0.003	[−0.007, 0.005]	0.74	0.01	0.01	[−0.01, 0.04]	0.27	9.61×10^{-3}	0.004	[0.001, 0.02]	0.03
Maternal age at birth	7.67×10^{-3}	0.004	[−0.001, 0.02]	0.09	2.61×10^{-3}	0.009	[−0.02, 0.02]	0.76	1.67×10^{-3}	0.007	[−0.01, 0.02]	0.82

[a] Cluster-robust standard errors can account for dependence of samples within matched pairs (7). [b] SE: standard error. [c] 95%CI: 95% confidence interval.
This table shows the association results including only samples with a “Pass” laboratory designation.

Table S5. Sensitivity analysis examining Trimester 3 DNAm data in the discovery (i.e., validation), replication, and combined samples of replication phase while including samples with "Pass" or "Check" laboratory designation (Table S3)

**Replication phase**	**$N/n_{control}/n_{case}$**	**N_{pairs}***	**Mean DNA methylation as beta-values (%)**		**Mean DNA methylation as M-values**		**Direction (Cases vs. Controls)**	***p*****
			Controls	Cases	Controls	Cases		
**chr6:111902626**								
Validation sample	53/28/25	25	72.24	78.93	1.41	1.94	+	9.53×10^{-5}
Independent sample	114/50/64	43	75.16	73.45	1.67	1.52	-	0.18
Combined sample	167/78/89	68	74.11	74.99	1.58	1.64	+	0.53
**cg16155413, chr6:111902611**								
Validation sample	53/28/25	25	46.20	56.58	-0.22	0.39	+	5.53×10^{-5}
Independent sample	108/48/60	39	53.06	48.35	0.18	-0.11	-	0.03
Combined sample	161/76/85	64	50.53	50.77	0.03	0.04	+	0.96
**cg21882990, chr6:111902385**								
Validation sample	53/28/25	25	78.55	80.85	1.94	2.11	+	0.28
Independent sample	114/50/64	43	76.82	76.91	1.77	1.78	+	0.87
Combined sample	167/78/89	68	77.44	78.01	1.83	1.88	+	0.54

*Number of matched pairs for which both observations were present; **P-values from the partially overlapping samples t-test; note the main analyses (Table 5), include only samples with a "Pass" laboratory designation. This table presents a sensitivity analysis including both "Pass" and "Check" samples.

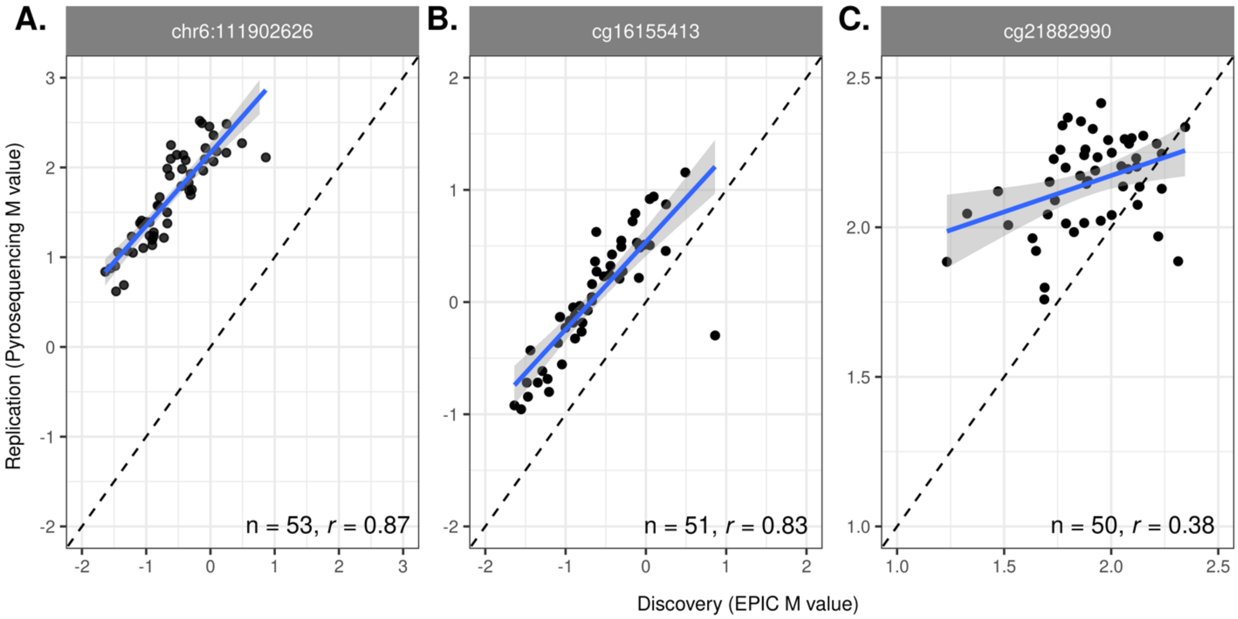

Figure S21. Comparison of DNA methylation M values for validation samples (i.e., overlapping samples between discovery and replication data). n is the number of samples and *r* is the Pearson correlation coefficient. The blue solid line is the regression line fitted to the data and the black dashed line is y=x.

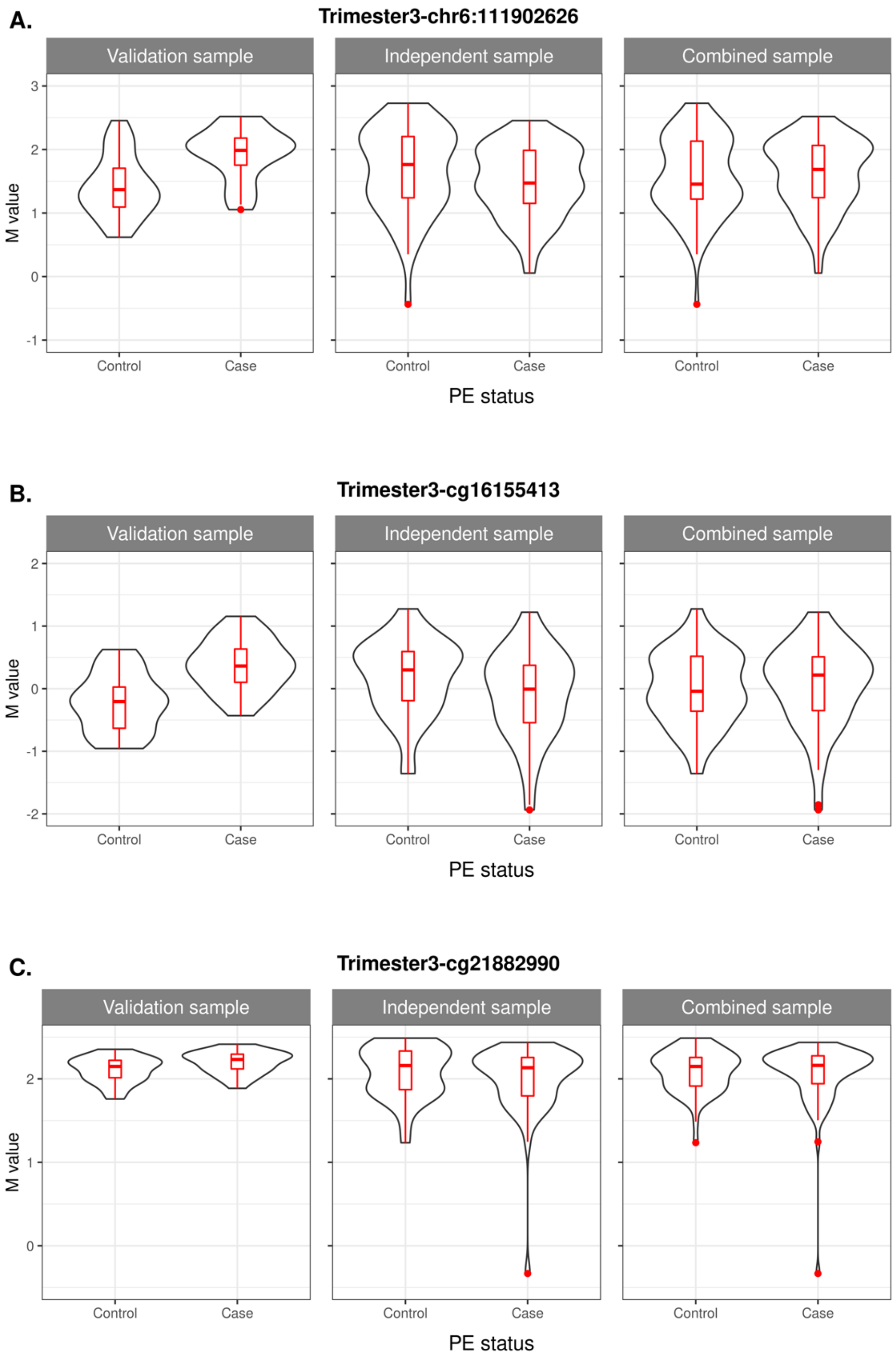

Figure S22. Distribution of DNA methylation M values between trimester 3 replication phase.
Validation sample, those with DNAm data from both the discovery (Infinium® MethylationEPIC Beadchip) and replication (pyrosequencing platforms); Independent sample, an independent sample of participants completely separate from the discovery phase; Combined sample, combination of validation and independent samples.

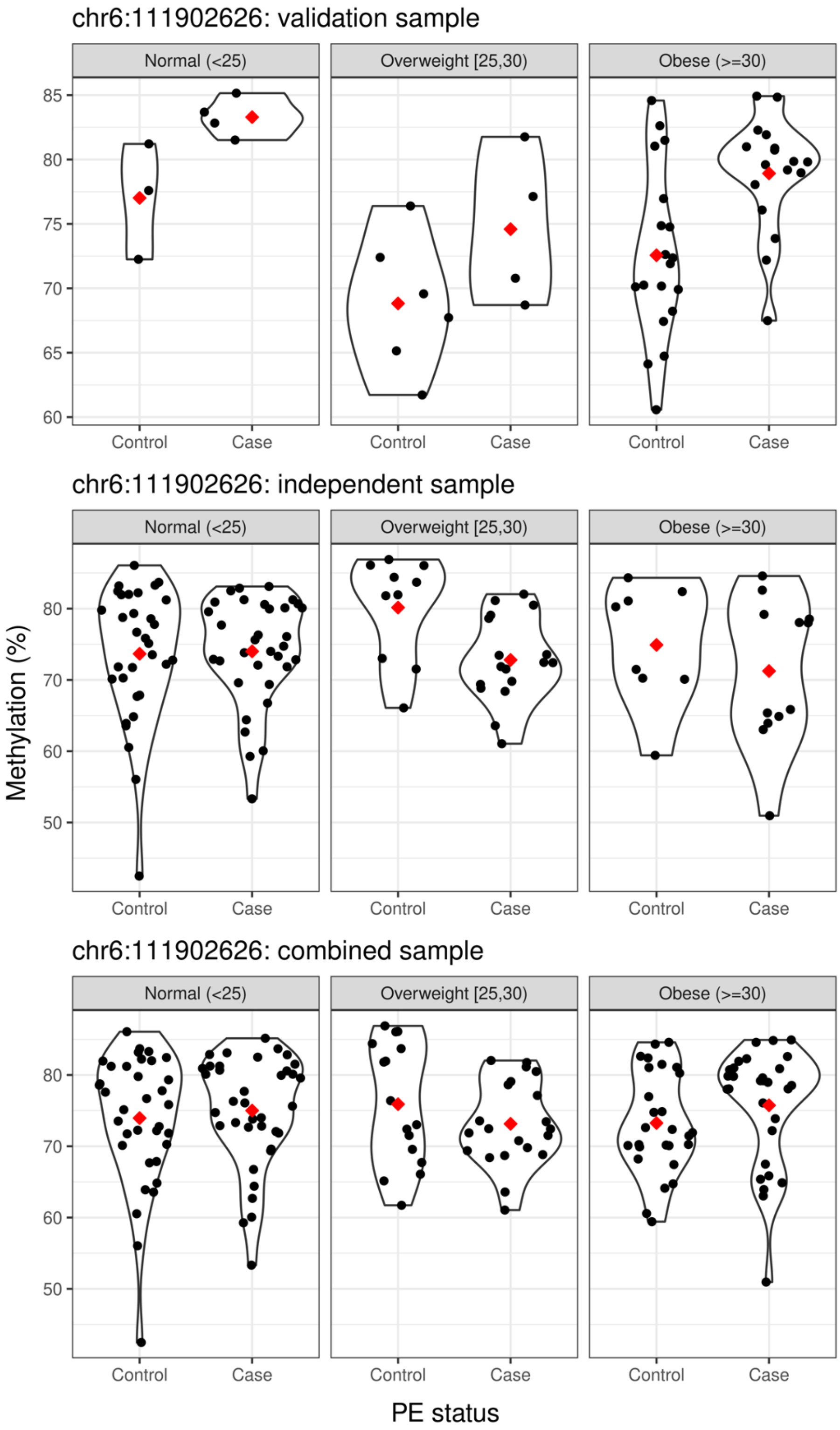

Figure S23. Distribution of chr6:111902626 DNA methylation level in three BMI groups. Red dot is the mean of DNA methylation levels.

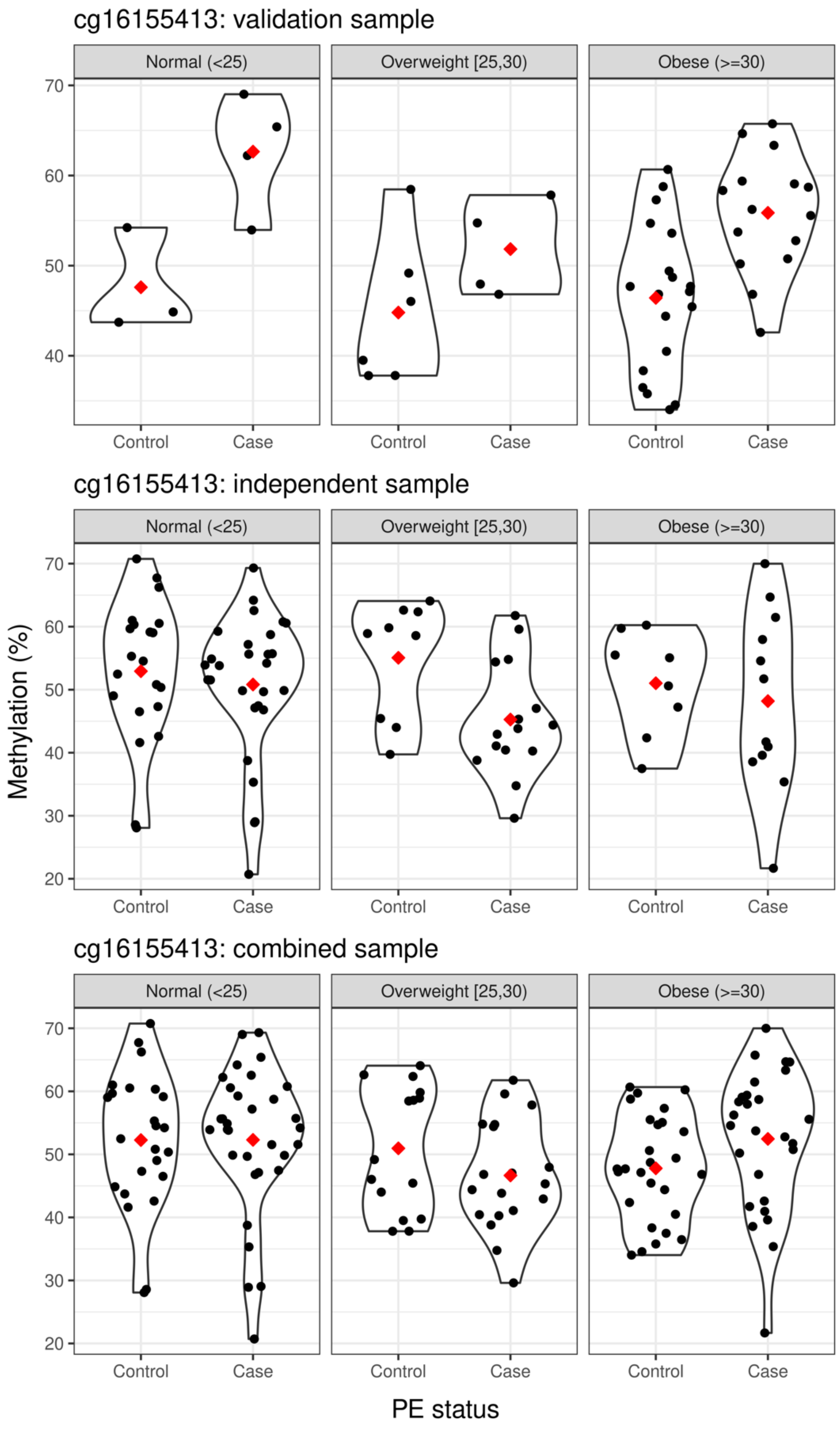

Figure S24. Distribution of CpG site cg16155413 DNA methylation level in three BMI groups.
Red dot is the mean of DNA methylation levels.

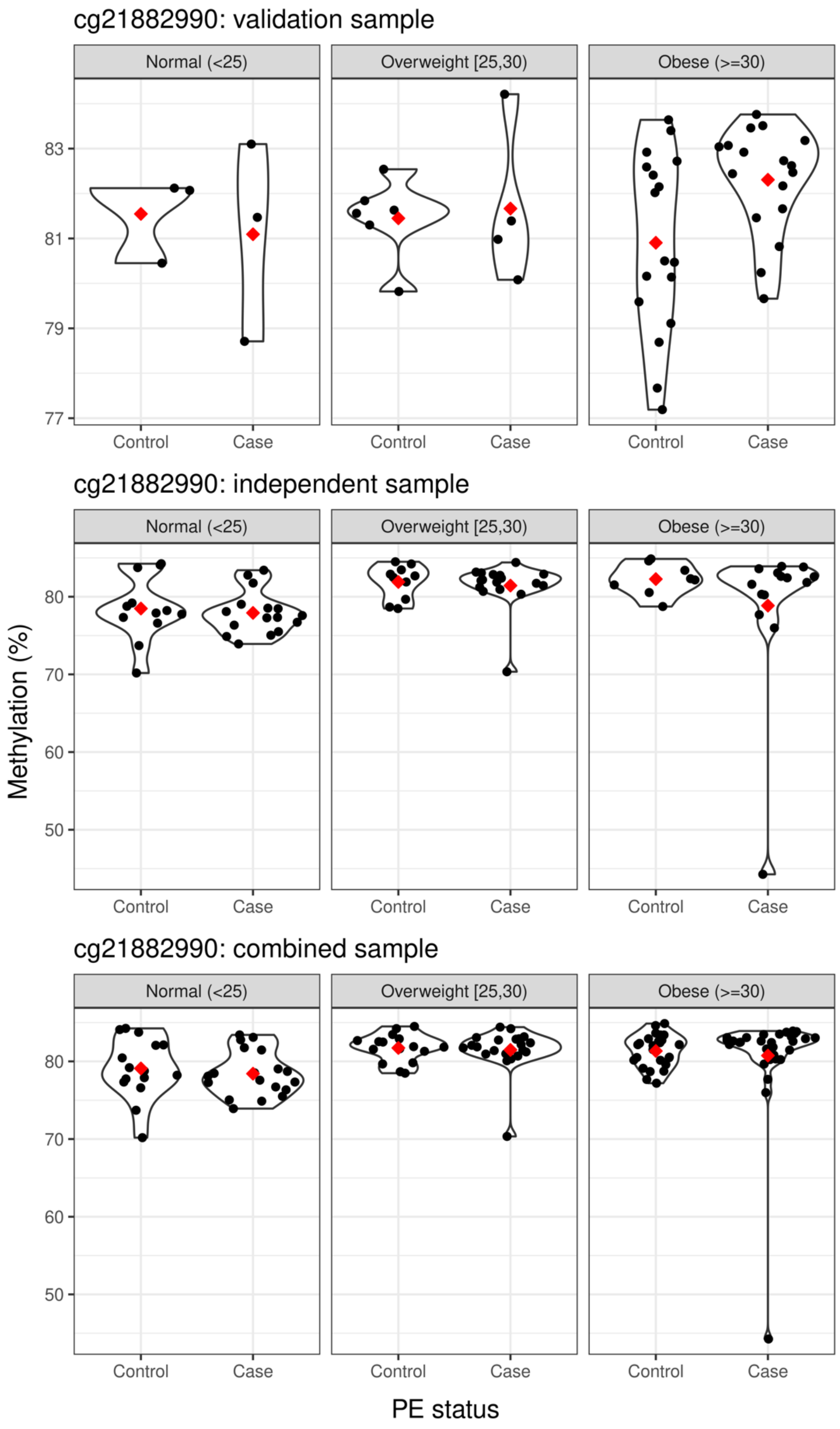

Figure S25. Distribution of CpG site cg21882990 DNA methylation level in three BMI groups.
Red dot is the mean of DNA methylation levels.

REFERENCES

1. Aryee MJ, Jaffe AE, Corrada-Bravo H, Ladd-Acosta C, Feinberg AP, Hansen KD, et al. Minfi: a flexible and comprehensive Bioconductor package for the analysis of Infinium DNA methylation microarrays. Bioinformatics. 2014 May 15;30(10):1363–9.

2. Xu Z, Niu L, Li L, Taylor JA. ENmix: a novel background correction method for Illumina HumanMethylation450 BeadChip. Nucleic Acids Res. 2016 Feb 18;44(3):e20.

3. Fortin JP, Triche TJ, Hansen KD. Preprocessing, normalization and integration of the Illumina HumanMethylationEPIC array with minfi. Bioinformatics. 2017 Feb 15;33(4):558–60.

4. Xu Z, Niu L, Taylor JA. The ENmix DNA methylation analysis pipeline for Illumina BeadChip and comparisons with seven other preprocessing pipelines. Clin Epigenetics. 2021 Dec 9;13(1):216.

5. Du P, Kibbe WA, Lin SM. lumi: a pipeline for processing Illumina microarray. Bioinforma Oxf Engl. 2008 Jul 1;24(13):1547–8.

6. Oros Klein K, Grinek S, Bernatsky S, Bouchard L, Ciampi A, Colmegna I, et al. funtooNorm: an R package for normalization of DNA methylation data when there are multiple cell or tissue types. Bioinformatics. 2016 Feb 15;32(4):593–5.

7. LIANG KY, ZEGER SL. Longitudinal data analysis using generalized linear models. Biometrika. 1986 Apr 1;73(1):13–22.
